# Data set on the effect of training and development on creativity of academic staff in a selected Nigerian university

**DOI:** 10.1016/j.dib.2018.03.025

**Published:** 2018-03-12

**Authors:** Maxwell Olokundun, Hezekiah Falola, Odunayo Salau, Stephen ibidunni, Fred Peter, Taiye Borishade

**Affiliations:** Covenant University, Nigeria

**Keywords:** Training and development, Creativity, Academic staff, Nigeria

## Abstract

This article presented data on the effect of training and development on academic staff creativity using Covenant University in Nigeria as the case study. The article was a based on a descriptive quantitative research design using Survey method. The population of the study included all academic staff in the selected university with a total of 535 faculties. A sample size of 226 faculties was selected. Reliability and validity procedures were confirmed. Data was analyzed with the use of Statistical Package for Social Sciences (SPSS).Regression analysis was employed as statistical tool of analysis. The field data set is made publicly available to enable critical or extended analysis.

## 1 Introduction

Many university students in Nigeria are not able to obtain the information and support they need to persevere and graduate which earn institutions a reputation as being unsympathetic to student needs [1]. This owes largely to the absence of training and development of academic staff of these universities. Undoubtedly, training and development programmes for academic staff have long-term profits that outweigh the immediate costs. Coordinated training and development of academic staff is important because all students, irrespective of their courses deserve to have access to teachers or instructors who are knowledgeable and up-to-date on the policies, procedures, theories, and resources that help students thrive [4]. People want to feel knowledgeable at their jobs; hence providing academic staff with training and professional development can boost creativity in job performance [5]. Neglecting training and professional development for academic staff affects not only students, but also the institution which bears the costs of reduced efficiency. Therefore, this article presents data on the effect of training and development on creativity of academic staff in a Nigerian University in a bid to motivate critical or extended analysis on the subject.

**Specification Table**

**Table:** 

**Subject area**	Business, Management
**More Specific Subject Area:**	Human Resource Management
**Type of Data**	Table
**How Data was Acquired**	Researcher-made questionnaire analysis
**Data format**	Raw, analyzed, Inferential statistical data
**Experimental Factors**	Sample consisted of Academic staff. The researcher-made questionnaire which contained data on training and development and creativity of academic staff were completed..
**Experimental features**	Creativity is an important component of employee performance in contemporary organizations
**Data source location**	South west Nigeria
**Data Accessibility**	Data is included in this article

## Value of data

These data present information on training and development as it relates to creativity of academic staff in the university context. This is important considering that training programmes strengthen those skills that an employee needs to improve, while a development programme brings employees to a higher level so they all have similar skills and knowledge.

The results showed that organizing and facilitating training and development for academic staff can expedite acquisition of creative skills required for effective job performance.

The results can motivate the identification of relevant training and development regime required to stimulate creativity in academic staff.

## 2 Data

The data comprised raw inferential statistical data on the effect of training and development on creativity of academic staff of covenant university Nigeria. Explicitly, regression analysis was used to test the effect of the independent variable on the dependent variable. Table 1 shows the model summary of the analysis based on the hypothesis tested. The Model summary’ table provides information about the regression line's ability to account for the total variation in the dependent variable.

H_**01**_ Training and development does not affect employee's creativity.

**Table 1:** Model summary.

Model	R	R square	Adjusted R square	Standard error of the estimate
1	.550^a^	.303	.300	.483

a Predictors: (Constant), Training and Development

Table 2 is the Analysis of Variance Table which shows the statistical significance of the results obtained. The Analysis of Variance table compares the variance of the residuals from the regression model to the variance of the original data.

**Table 2:** Analysis of variance a.

Model		Sum of squares	Degree of freedom	Mean square	Frequency	Significance
1	Regression	21.421	1	21.421	91.737	.000^b^
	Residual	49.269	211	.234		
	Total	70.690	212			

a Dependent Variable: Creativity

b Predictors: (Constant), Training and Development

Table 3 is the regression coefficients. The regression coefficient provides the expected change in the dependent variable for a one-unit increase in the independent variable.

**Table 3:** Coefficients.

Model		Unstandardized coefficients		Standardized coefficients	T	Significance.
		B	Standard. error	Beta		
1	(Constant)	1.131	.269		4.204	.000
	Training and Development	.653	.068	.550	9.578	.000

^a^Dependent variable: Creativity

## 3 Experimental design, materials and methods

The data presented was based on a quantitative study. Descriptive research design was adopted to examine the effect of training and development on creativity of academic staff [2], [10]. Survey method was considered appropriate as data collection method. Covenant University was selected from South west Nigeria [9]. The study population included the academic staff of Covenant University, which has a population of 535 employees. 226 academic staff were selected to participate in this study. Data were collected from academic staff across the different colleges in the selected university using a researcher- made questionnaire [3], [6], [7], [8]. The collected data were coded and entered into SPSS version 22. Data analysis was done; using Statistical Package for Social Sciences-22. Data was analyzed using inferential statistical tests which involved regression analysis.

Although Statistical Package for Social Sciences may be limited when it comes to advanced modeling and development of statistical approaches however, for a less complicated data such as these, Statistical Package for Social Sciences makes in-depth data analysis quicker because the program knows the location of the cases and variables. It also comes with more procedures of screening the information in preparation for further analysis. More importantly, Statistical Package for Social Sciences is designed to make certain that the output is kept separate from data itself particularly because it stores all results in a separate file that is different from the data.

## 4 Conclusion and implications of the study

It is important to improve the training and development regime of academic staff in ways best suited for institutional culture and contexts in universities. This may boost the creative performance of academic which also has implications for institutional reputations. However, most importantly, training and development enhance students to acquire the necessary information, guidance, and support needed to learn, persist, graduate, and achieve their aspirations. Therefore, the data presented in this article is important in this regard for extended investigation or inquiry.
