# Supplementary material for: Data set on the effect of training and development on creativity of academic staff in a selected Nigerian university
Source: Data Brief. 2018 Mar 12;18:399–403. doi: 10.1016/j.dib.2018.03.025 (PMC5996224; doi:10.1016/j.dib.2018.03.025)
Supplement: Supplementary file 2 — Supplementary material [file mmc2.docx]

**MOTIVATION AND EMPLOYEE PERFORMANCE**

**CHAPTER ONE**

**INTRODUCTION**

**1.0 Background of Study**

The success of an organization or business depends to a great extent on the motivation of its workers. Motivation is the way to making and empowering a domain where ideal execution is conceivable. This prompts to the question how then do we guarantee that the individual motivation is at its peak inside the organization or workplace (Chapman, 2004). Motivation of workers is the key of a successful organization to keep up the congruity of work and to accomplish organizational goals and help the organization survive.

Each employee or worker would have his/her own particular arrangement of motivation that would drive him/her towards buckling down and performing proficiently. Motivation could be by acknowledgment or money impetuses. Whatever, the type of employee motivation, the way to advancing that motivation as an employer, is understanding and impetuses (Mc Coy, 2000).

Employee motivation is impression of duty, inventiveness and vitality that an organization's employee conveys to their occupations. However motivation can be characterized as an inward express that causes a person to carry on in a way that guarantees the achievement of a few goals. (Rudolph & Kleiner 2010). Garderner and Lambert (2012), characterized motivation as it is about the moving employees toward doing the occupation and accomplishing the objective through prizes. Moreover Hislop (2009), all around characterized motivation as a drive which pushes workers to do things which is a result of the individual needs being fulfilled so they have the motivation to finish and continue with the undertaking. Employee needs in the organization ought to be put one next to the other to the organizational needs. Motivation drives people/individuals to meet organizational goals through each test or imperative they may confront in their workplace (Hsee & Welch 2001).

Motivation attributes include; an inside constrain that initiates conduct, an objective which conduct is coordinated to accomplish and a maintained conduct. Motivation helps entrepreneurs and employees to be capable, ingenious and perform productively in day by day business errand. At the point when an employee is motivated, it builds activities, reinforces the aspiration, and gives strength, heading and diligence to accomplish/take after organizational goals. How employees feel about the organization can influence their execution and the solidness of the organization (Schwarz & Clore (2008). A temperamental organization fails to meet expectations.

Employees should be motivated to complete their potential. Some of which is the part of reward framework in motivating employees/workers in view of their extrinsic/external necessities, giving open doors that interest to their intrinsic/internal motivational needs. Festinger (2009). Motivation of workers is fundamental to an organization/business in other to lead an organization to elite rate/high performance.

**1.1 Statement of Research Problem**

This study is projected towards providing reasons on how motivation affects the performance of employees in an organization. The lack of motivation in an organization will lead to low staff turnover, low input level and poor attitude to work. Ryan (2012) said for and organization to triumph and be in harmony it’s from productivity and profitability to recruiting and retention, hardworking and happy employees. To meet certain standards in an organization/business employers find it challenging to motivate employees/workers to contribute voluntarily to the organizational performance. Studies such as Ubaka (2005) looked at the impact of employee’s motivation on organizational productivity. Pujari (2004) worked on the effect of employee motivation and retention ignoring the effects of motivation on employee performance.

Once an employee is highly compensated in an organization, it leads to the commitment of that employee to the organization. Compensation is a strong predicator of an employee’s commitment to an organization. An employee who is not appropriately compensated would cause a negative effect on how loyal or dedicated the employee would be to that organization. The lack of good compensation packages or strategies would affect the employee’s commitment to the organization. It also reduces the sense of loyalty an employee might have for the organization. Studies such as Kleon (2007), researched on the effects of compensation in employee’s performance and Dessler (2005) looked at the impact of compensation on the performance of an organization. However there is a need to look at how to determine the effects of compensation on employee commitment in a University setting.

The training and development of employees in the organization facilitates an employee’s creativity and the mental characteristics giving room for the employee to think outside the box resulting to innovative ideas and approaches to a particular task. Training and development programs are a key to enhancing the skills, abilities and knowledge of the employees. When employees are not trained or are not engaged in these training and development programs, these employees will not be prone or exposed to new ideas and will lack the ability to think wide or outside the box. The organization will face a limitation on the employee’s knowledge and competencies. According to Kabungaidze (2013) researched on the impact of training and development on employee retention in an organization. Mary (2012) did a study on the impact of training and development as a reward strategy for employee productivity. However there is a need to explore the extent to which training and development affects employees creativity in the University setting.

There is need for an employee to achieve equilibrium between primary priority of their employment position and their private lifestyle. If an employee is able to have a balance it will lead to a measure of efficiency in converting inputs into useful output. The work-life balance of employees greatly affects their disposition to work. There should be available flexi-working hours of the employees in the organization in other for the employees to be productive. The lack of a proper work-life balance of an employee would yield negative employee productivity. The absence of flexi-working hours will make an employee to be reluctant to work and not doing a particular task properly. The productivity of that organization will be highly affected; employees should be given the opportunity to balance their work and their private life adequately for effective employee productivity. Similar studies on the impact of motivation on workers’ productivity by Igbaji (2013) and work-life balance and its effects on employee commitment by (Mayor, 2008). There is a need to emphasis on the role of work-life balance on employee’s productivity in the University setting.

However, considering the connection between the relationship of motivated workers and the impact of motivation on employee’s performance, especially in organizations which these reviews have overlooked, is the degree to which motivation enhances employee’s performance for organizational objectives to be accomplished. Dhar and Wertenbroch, (2006). Employee motivation progresses working condition understanding and extended employee execution. This study would along these lines try to discover how motivation is pertinent in employee performance.

**1.2** **Objectives of the Study**

**General Objectives**

The primary aim of the study is to access the effect of motivation on employee’s performance. The study also helps in analyzing the kind of motivation approaches and theories, allowing the organization make decisions on the type of motivation approach to be used effectively.

**Special Objectives**

1. To determine the effect of compensation on employee commitment
2. To measure the extent to which training and development affects employee’s innovation/creativity.
3. To determine the role of work life balance on employees productivity.

**1.3 Research Question**

These are various questions addressing the research problem by achieving the research objectives. The study seeks to provide answers to the following questions.

1. To what degree is the effect of compensation on employee commitment?
2. To what extent does training and development affect employee’s innovation/creativity?
3. In what ways does work life balance impact employee’s productivity?

**1.4 Hypotheses of the Study**

**H_01_:** Compensation does not have effect on employee commitment.

**H_a1_**_:_ Compensation has effect on employees’ commitment.

**H_02_**: Training and development does not affect employee’s creativity.

**H_a2:_** Training and development affects employee’s creativity.

**H_03_:** Work life balance does not impact employee’s productivity.

**H_a3_**_:_ Work life balance has an impact on employee productivity.

**1.5 Scope of Study**

The scope of the study was limited to Covenant University, and the study was targeted to full time workers/employees. The study includes the challenges or issues faced by employers attempting to motivate their employees, and the employee’s views on the institutions motivation strategies/method. The study focuses on how motivation packages affected employee’s performance.

**1.6 Significance of Study**

The finding of this study is vital. It is to analyze and discover specifically the effect of motivation on employee’s performance. This study will be significant to:

**Management:**

This is to enable the management of the institution through this study to see ways/instruments to be used in motivating employees for better performance. The study shows the importance of effective motivation and utilization of manager’s ideas to innovate employee motivation. In the long run, it can help in achieving the goals of the institution. It would serve as reliable and convenient for the management.

**Employees:**

The result of this study provides solution to employee needs and focuses on the employee’s interest as well as weakness in other for the employees to be motivated. The motivation of these employees will enhance institutional activities and achievement of institutional goals.

**1.7 Research Methodology**

The study adopted the use of questioners as a methodological tool for data collection. Data for this research would be based on two (2) sources; primary and secondary. Copies of questionnaires would be used to obtain the primary data, directly from the fields. However secondary source can be solicited from articles, journals, and textbooks. Analyses from the primary and secondary sources are collected and forms basic information. The data analyses were conducted through the use of statistical packages for social science (SPSS).

**1.8 Operationalization of Research Variables.**

Examining the effect of motivation on employee’s performance has the following construct:

Dependent construct as: Employee’s Performance

Independent construct as: Motivation

The above statement is mathematically expressed as

Y=f(x)

Where Y= Dependent variable

X= Independent variable

F= functional variable

Y= Employees performance

X= Motivation

Therefore, from this equation employees performance is dependent on motivation.

This is expressed as EP=f (M)

Where EP=Y

M=X

The X and Y are broken down as follows:

Y= (y_1_, y_2_, y_3_……n)

Y_1_=Productivity

Y_2_= Innovation

Y_3_= Commitment

X= (x_1_, x_2_, x_3_…..n)

X_1_= Compensation

X_2_= Training and development

X_3_= Work-life balance

**1.9 Outline of Chapters**

The study comprises of five (5) chapters, which includes:

**Chapter 1**: Introduction/ Background of Study

This is the introduction of the case study, the summary of motivation. It explains the scope, significance, limitations, aims and objectives of the study. It also stated the problems associated with the study and the organization of the study.

**Chapter 2:** Literature Review

This shows the literature review of the study, the conceptual, empirical and theoretical frame work of the study. It contains the various models, theories and approaches of motivation. It includes the definition of motivation and performance and how motivation affects employee’s performance. It also analysis the gaps observed.

**Chapter 3**: Methodology

This shows the research strategy and the tools used to accomplish research objectives.it shows the method of data representation and analysis applied in the study. It illustrates the sampling and sample, the population area, data collection activities and data analysis method.

**Chapter 4**: Analysis and Result

It outlines the test hypothesis and presentation of data. It shows the research objectives and questions.

**Chapter 5**: Conclusion and Recommendation

It provides summary of the study concludes and draws recommendations, of research work including all findings as well as recommendation.

**1.10 Definition of Terms**

**Motivation:** is defined as human psychological characteristics that contribute to a person’s degree of commitment.

**Incentives:** is defined as supplemental reward that serves as a motivational device for a desired action or behavior.

**Compensation:** is the defined as sum of the direct benefits (salaries, allowances bonus and commission) and indirect benefits (insurance, pensions and vacations) that an employee receives from an employer.

**Training and development:** is defined as the official and ongoing activities within and outside an organization designed to enhance the fulfillment and performance of employees.

**Work-life balance:** is defined as a comfortable state of equilibrium achieved between an employee’s primary priorities of their employment position and their private life style.

**Productivity:** is defined as the measure of efficiency of a person, machine, factory, system etc in converting inputs into useful output.

**Innovation:** The way toward translating an idea or invention into a good or service that creates value or for which customers will pay.

**Creativity:** is defined as the mental characteristics that allow you think outside the box, which results in innovative approaches to a particular task.

**Commitment:** is defined as a state or quality of being dedicated to a cause, activity, etc.

**CHAPTER TWO**

**Literature Review**

**2.0 INTRODUCTION**

This chapter reviews literature for the study. It discusses the conceptual, theoretical and empirical framework of the study. The chapter identifies various theories by different scholars of motivation on employee performance. The conceptual framework is built on the various definitions and concept of the study. The theoretical framework structure is the exploration from past writing that characterizes a study’s center hypothesis and ideas. The theoretical framework limits the research question and helps researchers make speculations.

Theories are detailed to clarify, foresee, and comprehend marvels and in many cases, to challenge and amplify existing learning inside the breaking points of basic jumping presumptions. The theoretical framework is the structures that can hold or bolster a theory of an exploration contemplate. Swason (2013).The theoretical framework presents and depicts the hypothesis that clarifies why the research issue under review exists. Theoretical research is hypothetical. This chapter looks into ways motivation can affect the employee’s performance in an organization.

Empirical framework is observed and measured in view of marvels and gets learning from genuine encounter as opposed to from theory or conviction. Empirical research utilizes exact confirmation. Empirical research begins from particular solid illustrations/perceptions to make a model and in this way, a hypothesis/theory. Empirical research is in this manner inductive, i.e. it is base up, however not really so. It is a method for picking up information by method for immediate and circuitous perception or experience, (Toh Wie Min, 2016). There have been researches on the effect of motivation on job satisfaction, effect of motivation on employee commitment, but this research would focus on the gap between the effects of motivation on employee performance in Covenant University.

**2.1 Conceptual Framework**

**2.1.1 Concept of Motivation**

Motivation was coined from a word motive which means emotions, ideas and needs which drives an individual into action. A motive is what prompts the person to act in a certain way, or at least develop an inclination for specific behavior. Stimulus is behind however a man behaves. It is dependent on the individual’s motive. Motivation is a complex phenomenon, which is influenced by individual, cultural, ethnic and historical factors. Motivation can be defined as “a series of energizing forces that originate both within and beyond an individual’s self”. These forces determine the person’s behavior and therefore, influence his/her productivity (Jackson, 2010). According to Cenzo (2010), people who are motivated use a greater effort to perform a job than those who are not motivated.

Linder (2011) Defined motivation as “the psychological process that gives behavior purpose and direction, a predisposition to behave in a purposive manner to achieve specific unmet needs, an unsatisfied need and the will to achieve, respectively.

Young (2000) also defined motivation as the force within an individual that account for the level, direction, persistence of effort at work. Halepota (2005) definition of motivation is “a person’s active participation and commitment to achieve the prescribed results”. Halepota further presents that the concept of motivation is abstract because different strategies produce different results at different times and there is no single strategy that can produce guaranteed favorable results at all times. According to Latham and Ernest (2006) motivation was in the beginning of the 1900s thought only to be monetary.

Jones (2010) argues that “motivation is concerned with how behavior gets started, is energized, is sustained, is directed, and is stopped and what kind of subjective reaction is present in the organization while all this is going on.

**2.1.1.1 Types of Motivation**

According to Crabbe (2006), researchers have identified two primary sources of motivation. The intrinsic and the extrinsic forms of motivation. Every individual is different, and to effectively motivate each individual there must be an understanding on the types of motivation. With this understanding you will find out that some individuals respond best to intrinsic will others to extrinsic.

**2.1.1.2 Intrinsic Motivation**: Intrinsic motivation is defined as the doing of an activity for its inherent satisfactions rather than for some separable consequence. The individual has the desire to perform a specific task, because its results are in accordance with his belief system or fulfills a desire. The phenomenon of intrinsic motivation was first acknowledged within experimental studies of animal behavior, where it was discovered that many organisms engage in exploratory, playful, and curiosity-driven behaviors even in the absence of reinforcement or reward (White, 2011). This natural motivational tendency is a critical element in cognitive, social, and physical development because it is through acting on one’s inherent interests that one grows in knowledge and skills. Intrinsic motivators are concerned with the quality of work life, and are likely to have deeper and long term effect because they are inherent in individuals and are not imposed from outside (Armstrong, 2006).

Intrinsic motivation is related to psychological rewards such as using one’s ability. Psychological rewards are those that can usually be determined by the actions and behavior of the individual managers (Mullins, 2010). Intrinsically motivated behaviors are seen when there is no other apparent reward except the activity itself (Deci, 2011). Malone and Lepper (2009) have defined it as what people will do without external inducement.

Examples of intrinsic motivation are hunger, a sense of duty, altruism, and a desire to feel appreciated. Sentiments of achievement, social contact, social status, accomplishment test and ability got from performing ones work are examples of intrinsic motivation. Intrinsically motivated behaviors, which are performed out of interest and satisfy the innate psychological needs for competence and autonomy, are the prototype of self-determined behavior.

**2.1.1.3 Extrinsic Motivation:** Extrinsic motivation is a construct that pertains whenever an activity is done in order to attain some separable outcome. Extrinsically motivated behaviors are those where the controlling mechanism is easily seen Deci (2012). Examples of extrinsic motivation are money, rules and laws, employee of the month, benefit packages, bonuses and the physical environment. It is related to tangible rewards such as salary and fringe benefits, security, promotion, contract of service, the work environment and conditions of service.

Extrinsic motivators can have an immediate and powerful effect but will not necessary last long (Mullins & Armstrong, 2011). Akintoye (2008) asserts that money remains the most significant motivational strategy. Katz and Sinclair (2014) demonstrates the motivational power of money through the process of job choice. He explains that money has the power to attract, retain, and motivate individuals towards higher performance.

There are four (4) types of extrinsic motivation:

1. **External regulation:** Such behaviours are performed to satisfy an external demand or obtain an externally imposed reward contingency.
2. **Introjected regulation:** it describes a type of internal regulation that is still quite controlling because people perform such actions with the feeling of pressure in order to avoid guilt or anxiety or to attain ego-enhancements or pride.
3. **Identification:** Here, the person has identified with the personal importance of a behaviour and has thus accepted its regulation as his or her own.
4. **Integrated regulation:** Integration occurs when identified regulations have been fully assimilated to the self. This occurs through self-examination and bringing new regulations into congruence with one’s other values and needs.

Extrinsically motivated behaviors are those that are executed because they are instrumental to some separable consequence can vary in the extent to which they represent self-determination.

**2.1.2 Importance of Motivation on Employee Performance**

1. Motivation leads an organization to profitable operations. Every motivated employee will perform work or task with full responsibility, which leads to the utilization of resources.
2. It leads to the development of workers efficiency, this development contributes to maximizing production and productivity.
3. It encourages the effective use of human resources by leading motivated employees to make best utilization of their skills, knowledge and capability in the environment.
4. It helps to maintain coordination and develop a feeling of harmony between workers and management.
5. Motivated workers put their effort towards the attainment of organization objectives.
6. It creates confidence in the employees to get their needs satisfied in the organization. Employees turnover are less because the satisfied employees never leave the job.
7. Motivation will lead to optimistic and challenging attitude at workplace.
8. Employee motivation will lead to adaptability and creativity during amendments periods.

Motivation is a very vital factor on employee performance; this is because when employees are motivated appropriately they gained optimum satisfaction and tend to perform effectively in the organization. Motivated employees are usually committed to the organization which leads to less employee turnover in the organization.

**2.1.3 Principles of Motivation**

According to John Sylvester (2013), keeping employees motivated needs constant attention and commitment. Some of these principles include;

1. Holding regular staff or team meetings to keep your employees updated on developments, goals and targets of the organization through constant communication.
2. Rally team members to help fellow employees when an employee is stuck in a problem.
3. Getting to know your employees, knowing what motivates them and the kind of incentives they would choose.
4. Encourage participation by including your staff in team decisions where appropriate.
5. Flexible working schedules help the employees achieve a better work-life balance.

**2.1.4 Sources of Motivation**

Hitt (2009), adding to motivation was of the feeling that, there are fundamentally three classifications of factors that decide motivation at the work setting therefore;

Characteristics of the individual - the main class, are the wellspring of interior or push strengths of motivation. This he claims is the thing that the worker conveys to the work setting. Safeguarding his point additionally attests that three factors likewise add to an individual’s push constrains: the people (1) Need- for example, security, self-regard, accomplishment, or power. (2) Attitudes-towards work, a boss, or association and (3) Goals- for example, completion, achievement of a specific level of performance, and bearer progression.

Characteristics of the job-the second class as indicated by him, identifies with the outside or pull powers which move in job characteristics of the individual (what the individual does at the working environment). The characteristics he laid out as what amount coordinate criticism he gets, the work stack, the variety and scope of task and extent of undertakings and level of control the individual has as far as how he or she functions.

Characteristics of the work situation – the third class he recognized, and from his accommodation it plainly demonstrates that it identifies with the work situation of the individual, discussing what really happens to the individual. A further perusing by the researcher, uncovered that this classification has two arrangements of factors: the quick social environment containing the persons directors, working gathering individuals and subordinates; and the different sorts of organizational activities, for example, the organizations compensation practices, the accessibility of preparing and advancement, and the measure of weight connected to accomplish high amounts of output.

**2.1.5 Compensation**

Compensation is a contractual phenomenon. According to Belcher (2007), it’s a double input-output exchange between a worker and an employer. The input of efforts and output of wages are established. Stahl (2005), defines compensation as the monetary payment wages, salaries emoluments, bonuses both current and deferred, and used reward employees.

Casio (2008) defines compensation as including direct financial payments plus indirect payment in form of fringe benefits. The two definitions equate compensation with monetary income only. Compensation is defined by Mondy (2010) as the total of all rewards provided to employees in return for their service, the overall purposes of which are to attract, retain and motivate employees. Brown (2003) saw compensation as a return in exchange between the employees and themselves as an entitlement for being employee of an organization, or as a reward for a job well done.

Compensation is direct and indirect wages. Direct compensation includes wages, salaries, bonuses or commission based on performances, overtime work, holiday premium, while indirect compensation is paid as medical benefits, housing allowance, meal allowance, utility allowances, incentive bonus, shift allowances, hospitalization expenses, out of station allowance, vehicle loan benefits, annual leave allowances, car basic allowances, etc. The term “compensation” simply refers to the wages paid directly for time worked as well as more indirect benefits that employees receive as part of their job or employment relationship with an organization (Otobo, 2007). According to the American Compensation Association’s (2005) definition, “compensation is the cash and non-cash remuneration provided by an employer for services rendered” (ACA).

**2.1.5.1 Features of Compensation**

1. Compensation should be balanced including a reasonable combination of direct and indirect benefits of financial and non-financial reward.
2. Compensation should be based on merit and not favouritism.
3. Flexibility is a must in any compensation plan.
4. Machineries for resolving compensation issues must be clear to all.
5. Effective compensation system must focus on desired behaviour, reward positive work behaviour and sanction negative work behaviour.

Compensation is a form of motivation or a motivating strategy to improve employees’ performance in the organization. Compensation involves the entitlement or benefits given to an employee in an organization. A compensation package should not only be monetary but also non-monetary. It should be given based on merit, a negative behaviour of an employee should not be compensated but sanctioned and a positive behaviour of an employee should always be rewarded through the organizations compensation packages.

**2.1.5.2 Elements of Compensation Packages.**

1. **Basic pay:** is divided into salaries and wages. Salary is the fixed or guaranteed regular monthly or annual gross payment paid to employees. Wage is regular, it could be weekly or daily payment paid for work or services, usually to manual workers. According to Nwachukwu and Ogunbameru (2000) “wage and salary administration” refers to the development, implementation and on-going maintenance of a base pay system. The central objective or purpose of wage and salary administration is to provide pay that is both competitive and equitable (Atchison, 2003).
2. **Performance Incentives:** are rewards offered in addition to the base wage or salary and are usually directly related to performance. It serves as energizers, motivators and a drive to performance levels. Performance incentives are intended to reward measurable outcomes which result in or directly relate to the accomplishment of the organization’s mission and strategic objectives. (Gehart & Milkovich, 2012).
3. **Benefits:** these are supplementary in-kind reward made available to all employees of the organization. They are given to employees irrespective of their performances. Examples are staff bus, annual leave allowances, utility/dressing allowance etc. Nevin Adams and Dallas Salisbury (2005) defined benefits as the optional, non-wage compensation provided to employees in addition to their normal wages and salary.

**2.1.6 Training and Development**

Training and development is any attempt to improve current or future employee performance by increasing an employee’s ability to perform through learning, usually by changing the employee’s attitude or increasing his or her skills and knowledge (Chandan, 2005). Goldstein and Ford (1993) ‘training is defined as the systematic acquisition of skills, rules, concepts, or attitudes that result in improved performance in another environment. Therefore, training programs are planned to produce.

Campbell (2011) briefly differentiates training and development as training courses are typically designed for a short term, stated set purpose, while development involves a broader education for long term purposes.

**2.1.6.1 Importance of Training and Development**

According to Rao (2000) training and development is vital to employee’s performance. They include;

1. To remove performance deficiencies.
2. To cope with now technological complexity resulting from extension of operation to various regions of the country.
3. To prevent skill obsolescence.
4. To induce certain behavioral changes in employees.
5. To cope with technological advancement. Examples of mechanization and computerization.

Training and development gives both the organization in general and the individual representatives with advantages that make the cost and time a beneficial venture. Most employees have a few shortcomings in their working environment abilities. A training program permits you to reinforce those abilities that every employee needs to move forward. A development program conveys all employees to a more elevated amount so they all have comparative aptitudes and information (Bryan, 2012).

An employee who gets the vital training is better ready to play out her occupation. He/her turns out to be more mindful of well-being practices and appropriate techniques for essential assignments. The training may likewise assemble the employee's certainty since he/she has a more grounded understanding of the business and the obligations of his/ her occupation. (Frost, 2012).

**2.1.6.1 Types of Training Method**

According to Smriti Chand (2002), a large variety of methods of training are used in business to train different people in the organization. It is divided into two classifications:

1. **On the job Training:** Under these techniques new or unpracticed workers learn through watching associates or directors playing out the occupation and attempting to mimic their conduct. These techniques don't cost much and are less problematic as representatives are dependably at work, training is given on similar machines and experience would be on officially affirmed norms, and above all the trainee is learning earning. Some of the strategies are: coaching, mentoring, job rotation, apprenticeship and understudy.
2. **Of the job Training:** these methods are conducted in separate environment from the job or organization, materials needed for study are supplied. There is full concentration of learning and freedom of expression. Some of the methods are; Lectures and Conferences, Vestibule Training, Simulation Exercises, Sensitivity Training.

**2.1.7 Work-life Balance**

Clark (2000), defines work-family balance as “satisfaction and good functioning at work and at home, with a minimum of role conflict. It is generally agreed that work-life balance is important for an individual’s psychological well-being, and that high self-esteem, satisfaction, and overall sense of harmony in life can be regarded as indicators of a successful balance between work and family roles (Marks & MacDermid, 2006). However, there is a lack of consensus on how work-life balance should be defined, measured, and researched, and thus, the theorizing of what constitutes work-life balance, how it develops, and what factors enable or hinder it, is still in progress (Grzywacz & Carlson, 2007).

In the above conceptualization, work-life balance and imbalance are not seen as inherently beneficial or detrimental, respectively, for psychological well-being and quality of life. Instead, Greenhaus (2003) state that it should be empirically tested whether equal time, involvement, and satisfaction balance is better for an individual than imbalance in favor of either the work or family role. Work–life balance is a concept including proper prioritizing between "work" career and ambition and "lifestyle" health, pleasure, leisure, family and spiritual development/meditation. This is related to the idea of lifestyle choice.

**2.1.8 Concept of Employee Performance**

Employee performance is defined as whether a person executes their job duties and responsibilities well. Many companies assess their employee's performance on an annual or quarterly basis in order to define certain areas that need improvement. Performance is a critical factor in organizational success. According to Kotelnikov (2008), the extent to which employees are motivated in their work depends on how well those employees are able to produce in their job. He goes further, to assert that motivation is expected to have a positive effect on quality performance; employees who are characterized by a high level of motivation will definitely show higher work and life satisfaction. Employee performance is actually influenced by motivation because if employees are motivated then they will do work with more effort and by which performance will ultimately improve (Azar & Shafighi, 2013). According to the results of the study conducted by Yang (2008) on individual performance showed that performance of the individuals cannot be verified. Similarly he asserts that organizations can use direct bonuses and rewards based on individual performance if employee performance is notice able (Yang, 2008).

A worker derives a sense of self-worth in the process of performing the job and, upon completion of that job, a sense of accomplishment. Studies have shown that satisfied workers are more productive (Schermerhorn, 2003). Employee performance is not just tasks and work to be done just to get bonus or pay increase. It is an ongoing process where the main objective is to improve both individual and business performance Bake (2009). Employee’s performance is a process used in most organizations in order to determine the abilities and productivity of the employees and usually employees set their objectives and goals for the upcoming period; they do the best to achieve it, monitor it and develop the right skills (Vallerand, 2013).

In line with Yang (2008), Bishop (2007) investigated employee performance and revealed that acknowledgment and recognition and reward of performance of employees direct the discrimination between employee productivity. Moral and productivity of employees is highly influenced by the effectiveness of performance of an organization and its reward management system (Yazıcı, 2008). According to Seidenfeld (2007), evaluating worker performance and giving feedback to employees is an undertaking most bosses fear. Thus, they tend to put it off, or more terrible still, stay away from it all together. However giving feedback to employees is an essentially vital administration work.

**2.1.8.1 Productivity**

The concept of productivity is generally defined as the relation between output and input. (Singh, 2000). Productivity is commonly defined as a ratio between the output volume and the volume of inputs. In other words, it measures how efficiently production inputs, such as labour and capital, are being used in an organization to produce a given level of output. Productivity is considered a key source of economic/organizational growth and competitiveness s and, as such, is basic statistical information for many international comparisons and performance assessments (Paul Krugman, 2002).

Productivity in industrial engineering is the relation of output (produced goods) to input (consumed resources) in the manufacturing transforming process. Productivity is connected to the use and availability of resources. Therefore, productivity is reduced if there is lack of resources or no proper use of the organizations resources. Productivity is the measure of how efficient production inputs are such as labour and capital are being used in an economy/organization to produce a given level of output.

**2.1.8.2 Innovation**

This is process of translating an idea or invention into a good or service that creates value or for which customers would pay. Innovation can be defined as something original and more effective and as a consequence new that breaks into the market or society (Frankelius, 2009). Innovation is also viewed as the application of better solutions that meet new requirement, unarticulated needs, or existing market needs. (Maryville, 2001).

Innovation is applied creativity. Blohowiak (2000) says everyone is fond of innovation and management’s task is tap organizations bottomless reservoir of creativity. Managers must channel the ideas to ensure they are put to work. He also sees innovativeness as a process of, encouraging creativity and putting the resulting new ideas to work, to the developing opportunity. According to Bhasin (2012) innovation is intentionally bringing into existence something new that can be sustained or repeated which has some value or utility.

Innovation at the level of an individual firm might be defined as the application of ideas that are new to the firm, whether the new ideas are embodied in products, services, processes or in work organization, management or working system.

**2.1.8.3 Commitment**

The concept of commitment implies an enhancement of the individual and his or her skills, and not simply what this can deliver to the organization (Hendry, 2004). Commitment is believing in a goal and wanting to achieve it. Commitment is when an employee continues to work in that job and feels psychologically bound to the organization, regardless of whether it is fulfilling or not (Iles, 2005). A committed employee increases value to that organization and has tendency to be more determined in their work. Committed employees show positive behavior towards the organization.

**2.1.8.3.1 Components of commitment**.

According to John Meyer and Natalie Allen (2004) there exist three (3) component model of commitment.

1. Affective commitment: describes how much an employee wants to stay in an organization. An employee who is affectively committed is satisfied with their work; feels valued and are great assets to the organization.
2. Normative commitment: this relates to how much employees feel they should stay at their organization. Normatively committed employees feel leaving their organization would be disastrous and would create a void in knowledge and skills in the organization.
3. Continuance commitment: describes how much employees feel the need to stay in the organization due to lack of work alternatives and remuneration. An employee feels the need to stay in an organization because there would not be an improvement in their salary and fringe benefits if they move to another organization.

Henry David Thoreau (2006) advised; do not hire a man who does not work for money, but him who does it for the love of it.

**2.1.9 Training and development on employee innovation**

Training and development increases employee’s efficiencies innovation, invention, capacity to accept new techniques and technology (Mc Namara, 2008). Innovation is a core value and a competitive requirement which is an essential growth factor that can be stimulated through training and development initiatives. The critical method for sustaining innovation in organizations is the ‘’people’’.

Employee innovation has been defined as the intentional behaviour of an individual to introduce and/or apply new ideas, products, processes, and procedures to his or her work role, unit, or organization (West & Farr, 2010). Employee innovation is the new ideas, products, processes, and procedures being introduced or implemented do not have to be absolutely new to the field.

In other to utilize tools, techniques and methods there is the need for the people’s creative brain to function at full capacity. Vehar (2006). Training and developing programs are necessary in energizing the attitudes and behaviour of people in the organization which leads to high leadership skills and transforms the way they think.

Through training and development initiatives employees develop skills and creativity which improves their flexibility in adapting to change. Training and development Initiatives can stimulate inventiveness and improve a company’s competitive edge (Clapon, 2010). A training and development program is the best way to increase employee innovation and creativity to improve employee performance in the organization.

In the Nigerian context training and developing employees in the organization will increase and improve the method in which employees think in the organization. New ideas and more solutions to problems in the organization will be initiated due to the learning process through training and development. When employees in the organization are trained frequently there would be room for innovation or creativity leading the organization towards a competitive edge.

**2.1.10 Compensation on Employee Commitment**

Compensation has been defined in many terms by various scholars, but in this research, compensation are which include i.e. typically money awarded to someone as a compensation for injury, loss or suffering, money received by an employee from an employer as a salary or wages, or do/giving something to someone in return. Sing and Vinnicombe (2010). Compensation is not only in form of money but also in non-cash form, benefits such as pension, life and health insurance and retirement plans. Money is still the primary incentive used to lure employees in an organization.

Employee commitment is when an employee believes his/her future is tied to the organization and their willingness to make personal sacrifices for the organization (Blau, 2010). Employee commitment refers to the psychological attachment of employees to their workplaces (Meyer 2011).Commitment to worthwhile objectives evokes moral motives that can foster satisfaction even in the absence of economic or relational benefits (Meyer & Parfyonova, 2010). Compensation offers an opportunity for security, autonomy, recognition and improved self-worth (Hoyt Gerdloff, 2012).

Compensating employees in the organization is important in other to encourage employee commitment. If employees in the organization are satisfied with how the organization practices compensation policies, they would remain committed to the organization. Organizations objectives and work would not be effective in the competitive world and perform at peak level unless each employee is committed to the organization.

When a compensation package is viewed as not being fair inconsistent and inequitable, the compensation package fails to secure the employees commitment. “Hancock (2013), stated that the main predictor of turnover and performance hence organizations that are concerned about employees’ job commitment and want to keep high performing employees should consider increasing commitment. This requires job commitment on the part of all employees which can only be achieved through better management practices in the area of managing rewards and compensation, by creating a perception of fairness through competitive wages and benefits (Coetzee, 2005).

As organizations are faced with ever increasing competition and as they prepare for new challenges reward and compensation management plays a vital role as key component of survival in attracting motivating and retaining the best employees which ultimately lead to organizational efficiency and effectiveness thus realizing high productivity and profitability Malkovich & Newman (2010).

Braggs (2009), argue that companies with committed employees will outperform companies with uncommitted workforce and contents that demonstrating commitment to employees will generate a reciprocal commitment by employees.

In the Nigerian context compensation on employees’ commitment is important in retaining the employees in the organization. A satisfied employee would be committed to the organization and will offer sources that will to organizational competence. Employees’ commitment to the organization is highly influenced by the compensation and reward system; organizations should have the best approach in other to enhance employee job commitment.

**2.1.11 Work life balance on employee productivity**

The idea of work-life adjust depends on the possibility that paid work and private life ought to be seen less as inverse needs and more as comparing basics of a full life. Work-life balance is the individual perception that work and non-work activities are compatible and promote growth in accordance with an individual’s current life priorities. (Kalliath & Brough, 2008).

Employee productivity is the amount of goods and services that a worker produces in a given amount of time (Fleck, 2009). Employee productivity is the amount of units of a product or a service that an employee handles in a defined time frame. Employee productivity (sometimes referred to as workforce productivity) is an assessment of the efficiency of a worker or group of workers.

Voydanoff (2008) posit that work-life balance is the global assessment that work and family resources are sufficient to meet work and family demands such that participation is effective in both domains. Work-life balance is generally associated with equilibrium, or maintaining an overall sense of harmony in life (Clarke et al, 2004).Chow and Keng-Howe’s, (2008) discovered a lot of workers revealed that the more flexible their schedules, the greater their self-reported productivity. According to an American psychological organization 2009, 69% of employees report that work is a significant source of stress and 41% say they typically feel tense or stressed out during the workday.

Lack of family- friendly policies, flexible schedules, Job design, and parental leave are stressing out many employees, reducing their job performance and productivity as well as causing broken homes (De Bruis & Dupuis, 2004). Organizations should formulate leave programs such as sick leave, maternity leave and paternity leave, which encourage employee productivity. Lack of flexible working schedule influences employees’ productivity negatively. To enhance work life balance it includes flexible working schedules and leave programs to determine employee productivity (Kalliath & Brough, 2008). Flexible working schedules are very critical in positively influencing employee productivity in the organization.

Work life balance is important to the organization and employees because when employees struggle to balance their work and family lives, their families and work will be negatively affected (Gryzwacz & Carlson, 2007). Clark (2002) defined work life balance as “satisfaction and good functioning at work and at home with a minimum of role conflict”. It could be called a condition of equilibrium where the demand of a person’s work equals that of his personal life. Delecta (2011), discussed that scholars’ work on work life balance explain the concept as the ability to satisfy three basic domains of life i.e. work, family and personal.

In the Nigerian context work life balance influences employee productivity in the organization positively or negatively. Once the working life of an employee is busy it affects the organization negatively due to stress and also affects the family and personal life of that employee. The organization should create leaves and flexible working hours for their employees in other to enhance work in the organization and increase employees’ productivity. The less stressed employees are the more effort the put into the organization which leads to productivity and effectiveness in the organization.

**2.2 Theoretical Framework**

**2.2.1 Motivation Theories**

Basic hunt of literature on the topic conveyed to the fore that, few theories of motivation have been produced, and were especially significant for work settings. In any case, the most intriguing disclosure was the negligible certainty that, each of these theories highlights at least one of the factors of motivation. Notwithstanding, it was additionally important to recognize that every one of these theories were propounded by American analyst/researcher.

**2.2.1.1 Abraham Maslow’s Hierarchy of Needs.**

This theory was propounded by Abraham Maslow. It depended on the suspicion that employees are inspired by arrangement of five widespread needs, these scope of needs he asserted the individual will be persuaded to satisfy whichever is most capable at the desperate hour (Maslow, 1970). This need, writing makes us to comprehend he assembled them into; Lower arrange needs-which he guaranteed are dominant until they are in any event somewhat fulfilled. From this edge it can be understood that any typical person would turn his thoughtfulness regarding fulfill needs at the following level offering ascend to higher-arrange needs which step by step gets to be distinctly dominant. Maslow (1943), expressed that individuals are motivated to accomplish certain needs and that a few needs outweigh others.


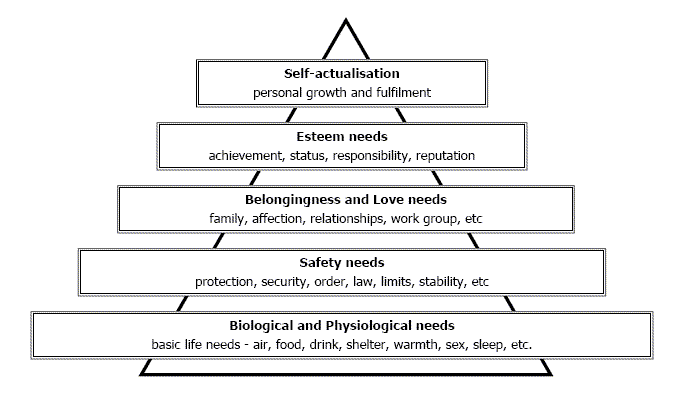


**Source: Author’s Own Construct, June 2012**

**2.2.1.1.1 Biological and Physiological Needs**.

Physiological needs as indicated by Maslow (1970), are the essential needs for survival and esteemed it to be the most minimal level needs. These needs included needs, for example, food, water and shelter. These are the fundamental necessities a person needs to survive and in actuality can't manage without it. He was of the solid supposition that regardless of the possibility that the various needs are unsatisfied then physiological needs will dominate. As long as physiological needs are unsatisfied, there exist as a driving or motivating power in a person’s life. A hungry individual has a felt need and this felt need touches off both (psychological and physical) strain and show itself in a way coordinated towards lessening the said pressure (inspiring sustenance to eat) accordingly, individuals will concentrate on exercises that will help them survive. Once the yearning is fulfilled pressure is lessened and the requirement for sustenance stops to be a motivator. Physiological Needs are the essential needs that each individual needs to survive. They are food, air, shelter and water. On the off chance that these fundamental needs are not met, then the employee won't have the capacity to experience different levels (Mary & Ann, 2011).

**2.2.1.1.2 Safety needs.**

The following level in the hierarchy was what he named as safety needs-the scan for shelter, security, steadiness, dependency, protection, flexibility from (nervousness, fear and confusion), and a requirement for structure, request, and law. Safety needs incorporate requirements for safety in one's physical surroundings, steadiness, and flexibility from emotional misery In the work setting this needs convert into a requirement for no less than an insignificant level of employment security; and the affirmation that we can't be rejected or sacked on irrelevant issues and that suitable levels of effort and profitability will guarantee proceeded with employment. Safety needs are the necessities related the feeling the employee of being secure and safe like having the medicinal protection, professional stability etc.

**2.2.1.1.3 Belongingness and Love needs.**

These needs are identified with the association with others. The relationship of the person with the general population environment. Like having companions and feeling acknowledged from others. As indicated by Hayes (2009), if a man has the initial two levels of necessities very much satisfied, the development of social needs (feeling of having a place and love) turns into the following target. At this phase in life, a man long for the fondness of others and would need to be put in a gathering or family. Relating this to the work put, as active creatures, people have a need to have a place and this must be fulfilled by a capacity to associate with one’s partners and have the capacity to team up adequately to accomplish organizational objectives. Belongingness need relate with longings for fellowship, love, and acknowledgment inside a given group of people.

**2.2.1.1.4 Esteem Needs**

Maslow (1970), saw from the research led with his patients that people in the wake of satisfying social needs would now long for what he calls esteem needs – in this manner, the craving for sense of pride, self-respect, esteem, and the esteem of others. Self-respect he characterized as the requirement for a feeling of (accomplishment, fitness, adequacy and certainty). Processing his submission deliberately, and relating it to the working environment setting, remotely, individuals look for needs like longing for reputation and acknowledgment, esteem, achievement, status, popularity, glory, predominance attention and appreciation according to other individuals.

Esteem is the feeling of being vital. Esteem needs are ordered to internal and external needs (Vance & Pravin 1976). Internal esteems are these identified with self-esteem like regard and achievement. External esteem needs are those, for example, economic/social status and acknowledgment that accompany the achievement. Esteem needs are those related with getting the regard of one's self as well as other people.

**2.2.1.1.5 Self Actualization**

The highest need in Maslow’s hierarchy, doubtful however. Self-actualization alludes to the craving for self-fulfillment, realization of a potential, constant self-development and the way toward getting to be you. Elaborating further, Hitt (2009), included that at this level, singular contrasts are dominant as the development of these necessities rest upon some earlier fulfillment of the past four. The researcher watched that, now, individuals trying to fulfill this need will really, search for individual relevance and esteem new responsibilities that may help find new talents.

It is the need of achieving the maximum capacity as an employee. As indicated by Vance and Pravin (2006), this need is never completely accomplished. Self-Actualization needs resemble truth, insight and equity. Providing a testing job with by one means or other intriguing angles will improve the self-actualization needs of the employee (Garderner & Lambert, 2012.) self-actualization needs are those comparing to the accomplishment of one's own potential, the practicing and testing of one's imaginative limits, and, when all is said in done, to turning into the best individual one can be.

A critical look at the theory where the researcher differentiates human needs in hierarchy. This is due to the fact that human needs are complex in nature, making them flexible in their method of doing things. Organizations can satisfy their employees by using money as a motivating factor to satisfy the safety and physiological needs. Organizations can provide entertainment activities to build good relationships between employees from different levels satisfying employees’ belongingness/love needs (Bradley, 2003).

Recognizing and rewarding employees will enhance their self-esteem. Satisfying employees need from the lower level which is the physiological and security needs should be met before upper level belongingness, esteem and actualization needs. Providing a safe working environment or condition, adequate pay, increased job responsibility, status, including challenging work in the organization will lead to an effective performance of the employees in the organization. They have no pressing need, however they are satisfied and can concentrate at work, which improves their performance and leads their organization to achieving its goals and objectives.

**2.2.1.2 Expectancy Theory**

The expectancy theory was propounded by Victor Vroom a therapist in 1964. The theory is just pertinent to a work setting that depends on people’s expectations. Vroom through these standards looked to depict that motivation is a component of the relationship between; effort exhausted by an individual and saw level of performance; and the desire that reward for coveted results will be identified with performance. Then again there must likewise be the desire that prizes are accessible as it will go far decide the quality of the motivational connection in this manner, the quality of the people preference for a result and the conviction that in the likelihood those specific activities will accomplish the required objective.

The expectancy theory in view of these assumptions has three key components: expectancy, instrumentality, and valence. An individual is motivated to the extent that he or she trusts that (an) effort will prompt to adequate performance (expectancy), (b) performance will be compensated (instrumentality), and (c) the value of the reward is exceedingly positive (valence). Effort →Required performance → Desired outcome

Force = Valence × Expectancy.


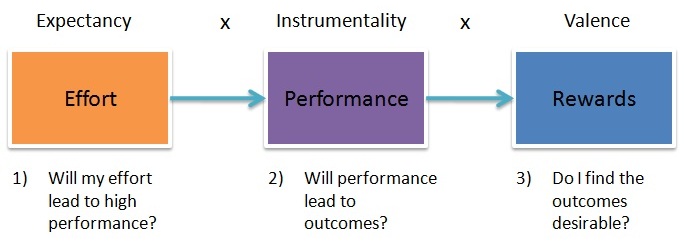


**Basic expectancy model**

1. **Expectancy:** is an individual’s gauge of the probability that employment related effort, will bring about a given level of performance. Expectancy depends on probabilities and extents from 0 to 1. If an employee sees no possibility that effort will prompt to the wanted performance level, the expectancy is 0. Then again, if the employee is totally sure that the errand will be finished, the expectancy has an estimation of 1.
2. **Instrumentality:** is an individual's gauge of the probability that a given level of accomplished undertaking performance will prompt to different work results. Similarly as with expectancy, instrumentality ranges from 0 to 1.if an employee sees that a decent performance rating will dependably bring about a compensation increment, the instrumentality has an estimation of 1. In the event that there is no apparent relationship between a decent performance rating and a compensation increment, then the instrumentality is 0.
3. **Valence:** is the quality of an employee’s preference for a specific reward. Therefore, compensation builds, advancement, peer acknowledgment, recognition by supervisors, or some other reward may have pretty much an incentive to individual employees. Unlike expectancy and instrumentality, valences can be either positive or negative. On the off chance that a worker has a solid inclination for achieving a reward, valence is certain. At the other outrageous, valence is negative. What's more, if an employee is not interested in a reward, valence is 0.

Motivation = Expectancy x Instrumentality x Valence.

**2.2.1.2.1 Effort-Performance Expectancy**

Leaders ought to attempt to build the conviction that employees are equipped for performing the job effectively. Methods for doing this include: select individuals with the required abilities and information; give the required preparing and elucidate work necessities; give adequate time and assets; appoint continuously more troublesome errands in light of preparing; take after representatives' recommendations about approaches to change their occupations; intercede and endeavor to alleviate issues that may impede successful performance; give cases of employees who have aced the assignment; and give coaching to workers who need self-confidence. Fundamentally, leaders need to make the craved performance achievable. Good leaders not only make it clear to employees what is expected of them but also help them attain that level of performance.

**2.2.1.2.2 Performance- Reward Expectancy**

Leaders ought to attempt to expand the belief that great performance will bring about esteemed rewards. Methods for doing as such include: measure job performance precisely; depict obviously the rewards that will come about because of successful performance; portray how the worker's rewards depended on past performance; give cases of different employees whose great performance has brought about higher rewards. In essence, leaders ought to connect specifically the particular performance they craving to the rewards coveted by employees. It is vital for employees to see plainly the reward procedure at work. Concrete acts must go with proclamations of goal.

Compensation systems can be a capable motivating force in connecting performance to rewards. Compensation frameworks that reward individuals specifically in view of how well they play out their jobs are known as pay-for-performance arranges (Berger, 2009). These may take such structures as "commission arrangements" utilized for deals staff, "piece-rate frameworks" utilized for assembly line laborers and field hands, and "motivating force investment opportunity (ISO) arranges" for officials (Dunn, 2009; Mercer, Carpenter, & Wyman, 2010) and different employees (Baker, 2011). Be that as it may, rewards connected to performance require not be monetary. Typical and verbal types of acknowledgment for good performance can be extremely successful too (Markham, Dow & McKee, 2002).

**2.2.1.2.3 Valences Reward**

Leaders ought to attempt to build the normal estimation of rewards coming about because of coveted performance. Methods for doing this include: convey rewards that employees esteem, and individualize rewards. With a demographically assorted workforce, it is misdirecting to trust that all employees seek similar rewards. A few employees may esteem advancement or an increase in salary, though others may incline toward extra excursion days, enhanced protection benefits, day care, or senior care offices. Many organizations have presented cafeteria-style advantage arranges—motivator frameworks that permit employees to choose their incidental advantages from a menu of accessible choices.

Another issue that may surface with anticipation hypothesis is the requirement for leaders to limit the nearness of countervailing rewards—performance rewards that have negative valences. For instance, gather standards (Mayo (2003). Roethlisberger and Dickson, (2009) may bring about a few employees to play out their jobs at least levels despite the fact that formal rewards and the job itself would somehow rouse them to perform at larger amounts.

Vroom's hypothesis gives a procedure of intellectual factors that reflects singular contrasts in work motivation. In this model, employees don't act basically in light of solid inward drives, neglected necessities, or the use of rewards. Rather, they are balanced individuals whose convictions, perceptions, and likelihood gauges impact their conduct. From a management outlook, the expectancy hypothesis has some essential implications for spurring employees. It distinguishes a few essential things that should be possible to inspire employees by modifying the individual's push to-performance expectancy, performance-to-reward expectancy, and reward valences. (Greenberg, 2011).

Taking a look at the theory and its complexity in nature one could just make recommendations to management, to truly attempt to discover craved result each of the associations values most, characterize the objective and what constitute the level performance expected to accomplish it. In any case, management ought to likewise not dismiss the way that the level of performance is reasonable, furthermore, achievable (Lawler, 2003). After a timeframe management ought to have the capacity to gauge the results by the performance craved by management. One thing significant is that the methodologies set up ought not to make struggle between the expectations it looked to make and other militating variables in the workplace. At last, results ought to be appealing and sufficiently tempting to touch off the coveted level of performance (Glinow, 2011).

**2.2.2 Employee Performance Theory**

**2.2.2.1 Reactance Theory**

Reactance theory became out of research on buyer conduct. This psychological theory depicts how individuals respond when they sense a risk to their opportunity of decision. The theory stretches out to numerous different parts of individual conduct that include motivation. Understanding reactance theory can help you motivate your employees and pick up their cooperation in more successful ways (Shelly Moore, 2010).

Researchers have discovered that telling individuals not to do something usually produces the opposite reaction. Individuals like to value their sense of freedom and display an image of self-control. Reactance theory proposes that, at whatever point individuals trust their flexibility either has or will be unreasonably threatened; they go into a reactance motivational state and act to recover control by not going along. Compulsion, specifically, prompts to the excitement of reactance, which thusly has a tendency to decrease compliance. (David Hanson, 2009).

According to reactance theory, when a man feels that his flexibility to pick an activity is limited, he will probably pick that specific conduct. Parent frequently encounter this at an early stage, finding that prohibiting an activity makes that activity additionally enticing to the youngster. Reactance drives individuals to play out the threatened or recently illegal conduct and demonstrate that despite everything they have choice. In a work environment, if an employee can't play out the conduct without taking a chance with his employment, he may begin accomplishing something else that is like the confined activity. He additionally may perform responsive practices, for example, taking additional long breaks or notwithstanding missing work days. (Brown Herald, 2008).

Reactance theory portrays the example of practices that happen in a person when they feel their freedoms are being taken away or restricted. This theory places that people trust they have certain freedoms and decisions and if these are threatened then contrary responses happen. At the point when practices that are seen as being free are threatened or taken away people can get to be distinctly motivated to hold and recover these freedoms (Ruth, 2011).

Jack Brehm (2007), projected that:

1. **Inclusion in Decision Making**

An employee usually wants to have some control over her employment and some freedom of decision at work. While employees generally react well when offered choices to help them advance and get extra rewards, they like to have some contribution to any choices that straightforwardly influence them, or if nothing else to be kept on top of it when the organization settles on these choices. Researchers considered associations that actualized another payment framework associated with performance; they found that examining the arrangement with the workers early prompted to enhanced performance. Not doing as such, interestingly, prompted to the arrangement being unsuccessful.

1. **Request Vs Demand**

A polite and balanced demand to employees tends to work superior to anything overwhelming enticing strategies or direct requests, notwithstanding when they know they must comply. Once more, employees need a feeling of having the capacity to pick their behaviors and to have some control over their occupations. For conduct you'd jump at the chance to see that isn't authoritatively commanded, utilizing an excessive amount of influence or solid arm strategies can drive the employee to oppose and even do the opposite you need.

1. **Supervision Consideration**

Employee conduct in circumstances where they needed to manage something the creators characterized as injurious supervision. According to reactance theory, employees baffled with excessively forceful supervision take part in retaliatory conduct, and also different sorts of degenerate conduct coordinated toward the manager as well as toward their partners and the association. The result of the review showed reactance theory is right in such manner.

**2.3 EMPIRICAL FRAMEWORK**

From the variety of conclusions of different authors an agreement or consensus can be arrived on the topic the effect of motivation on employee performance. A study done by Burney (2009), uncovered that the level of performance of employees depends on their genuine abilities as well as on the level of motivation every individual shows. Motivation is an inward drive or an outer instigation to carry on in some specific way, commonly a way that will prompt to rewards. Dessler and Harrington (2007), watch that over-accomplishing, gifted employees are the main impetus of all organizations so it is fundamental that establishments endeavor to motivate and clutch the best employees.

As indicated by Greenberg (2010) and Baron (2011), the meaning of motivation could be separated into three principle parts. The initial segment takes a gander at excitement that arrangements with the drive, or vitality behind individual(s) activity. Individuals swing to be guided by their enthusiasm for making a decent impact on others, doing intriguing work and being successful in what they do. The second part alluding to the decision individuals make and the course their conduct takes. The last part manages keeping up conduct obviously characterizing to what extent individuals need to persevere at endeavoring to meet their objectives.

Hitt Esser and Marriott (2004), indicated that motivation can be intrinsic and extrinsic. Extrinsic motivation concerns conduct impacted by getting outer prizes Praise or positive criticism, cash, and the nonattendance of discipline are cases of extrinsic or external prizes Deci (2009), contends that intrinsic motivation is the motivation to accomplish something just for the delight of playing out that specific movement. As indicated by Hagedoorn and Van Yperen (2005), cases of intrinsic elements are fascinating work, acknowledgment, development, and accomplishment.

Linz (2008), uncovered that few studies have found that there are certain relationship between intrinsic motivation and employment performance and also intrinsic motivation and occupation fulfillment. This is huge to establishments in today's profoundly focused business condition in that intrinsically motivated employees will perform better and, in this manner, be more beneficial, and furthermore in light of the fact that fulfilled employees will stay faithful to their organization and feel no weight or need to move to an alternate organization.

Deci and Ryan (2006), led and recreated an analysis that demonstrated the negative effect of money related rewards on intrinsic motivation and performance. A gathering of understudies were made a request to take a shot at an intriguing riddle. Some were paid and some were not paid for the work. The understudies that were not paid worked longer on the perplex and thought that it was more intriguing than the understudies being paid. At the point when the study was brought into a work environment setting, employees felt that their conduct was being controlled in a dehumanizing and distancing way by the prizes. It was found that prizes would truly diminish an employee’s motivation to ever play out the assignment being remunerated, or one like it, whenever later on.

Mulwa (2012), led a study on employee performance out in the open study establishments in Kenya. He clarifies the different motivation speculations like Abraham Maslow's chain of importance of requirements, Hertzberg's two component theories and the equity theory of Adams. The audit demonstrates that motivation is key for the efficiency, gainfulness and maintainability of each establishment - as the employees are its movers and its live blood.

Vroom (2007), suggests that individuals are motivated by the amount they need something and how likely they think they are to get it. He proposes that motivation prompts to endeavors and the endeavors joined with employees capacity together with condition components which interchange's subsequent to performance. This performance understudies prompt to different results, each of which has a related esteem called Valence.

Deci and Ryan (2011), in a joint effort with two of their associates led a study to inspect the impacts of performance unexpected rewards on a worker's intrinsic motivation. The review additionally found that these sorts of prizes are extremely controlling since these prizes are straightforwardly connected with a worker's performance of some undertaking (Koestner & Ryan, 2008).

Having communicated the complete of different researchers and saw the above communicated hypotheses , the course of this study is relatable to the above conclusions Therefore, a coordinating instrument conclusion from the checked on writing will be joined with disclosures from data clarification or understanding in chapter four to affirm the empirical discoveries in this study.

**2.4 GAPS IN LITERATURE REVIEW**

Studies such as Kleon (2007), researched on the effects of compensation on employee performance and Dessler (2005), looked at the impact of compensation on the performance of an organization. Only a few studies have researched on how to determine the effects of compensation on employee commitment in the University setting.

Few studies such as Kabungaidze (2013) and Mary (2012), have examined the impact of training and development as a reward strategy for employee productivity. However, there is a dearth of research on the extent to which training and development affects employee’s creativity in the University setting.

Researches such as Igbaji (2013) and Mayor (2008) have looked at work-life balance and its effects on employee commitment in the banking sector. Only limited research has been done to determine the role of work-life balance on employee productivity in the University setting.

**CHAPTER THREE**

**METHODOLOGY**

**3.0 INTRODUCTION**

The primary aim of this chapter is to recognize and clarify the techniques required in sourcing, translating information and breaking down data significant to this review. It is vital for one to comprehend the significance of the work to be finished by the researcher. Research is a methodical, formal thorough and exact process utilized to pick up answers for issues or potentially to find and translate new certainties and relationships (Waltz & Bausell, 2011). It is the way toward searching for a particular response to a particular question in a composed goal dependable way (Payton, 2009).

This chapter shows the research methodology and the techniques and also the legitimization of the decisions and their uses. Furthermore, the research procedure and design, study populace and setting, sample and sampling methodology, data accumulation, pilot study and data analysis strategy and administration. This chapter offers learning to the research methodology and in addition procedures used as a piece of relative motivation research.

This chapter additionally depicts research methods embraced, information gathering, studies, and questionnaire/poll, the data analysis strategies, the moral thought and ultimately the dialog of the validity and reliability of the study. This methodology concentrates on exact observation, perception and hypothesis confirmation. The analysts considered three procedures to coordinate the study including quantitative, qualitative and mixed methods. The level headed discussion among researchers on the best procedure to use for research has continued going during the time in light of the way that each method has its central focuses and drawbacks.

Be that as it may, in light of the research technique, the researchers watched and observed the mixed technique to be the most appropriate system for this study. The Primary data collection strategy constituted questionnaires. The methodological structure for this study and overview depends on quantitative analysis. Quantitative information gathering procedure has been utilized for the study. The fundamental research instrument was survey/questionnaire. This is to make it feasible for the accumulation of all types of data required. The questions were directed through the poll/questionnaire, which was given to the employees to react.

**3.1 RESEARCH DESIGN**

Research design includes a progression of discerning basic leadership decisions. The research design was conceived taking after some of the researchers choices related with the reason for the study. At the end of the day, the research design is the progression for designing the research study in a manner that the fundamental information can be accumulated and broken down to touch base at a solution (Sekaran, 2003). According to Akinade and Owolabi (2009), research design structures or ways a researchers plans to execute the study. It identifies with utilizing significant and all around developed surveys/questionnaires to gather the fundamental information. Coopers and Schindler (2006), likewise characterize research design as a blue print for the gathering, estimation, investigation of information and an arrangement to get answers to research questions.

The study utilized the quantitative research design. Quantitative research utilizes target estimation and factual investigation of numeric information to comprehend and clarify a phenomenon. The study used the descriptive survey design; data on the effect of motivation on performance was displayed as gotten from the respondent. Descriptive study is a procedure of gathering information with a specific end goal to answer questions concerning the current status of the subject in the study (Mugenda, 2003).The basis behind the choice of the design was that it helped the researcher to investigate the current status of motivation in the organization.

**3.2 RESEARCH METHODS**

Research methodology alludes to the arrangement, structure and procedure of examination considered on the premise against which claims for information are assessed (Ojo, 2003). Research methods are partitioned into different sorts. They incorporate; study look into technique, trial inquire about strategy and the time arrangement technique (Ojo, 2003). Survey method was used as data collection method for this study. Survey method was viewed as the most appropriate because it enhances the collection of data from a fairly large population (Ojo, 2003)

**3.3 STUDY POPULATION**

The population of the research, the total number of individuals that, the researcher wished to explore (Sekaran, 2003).A population alludes to the total of all cases that comply with some assigned arrangement of details it is the whole arrangement of important units of investigation or information (Kothari, 2008). A population comprises of all the possible components, subjects or perceptions that are of essential enthusiasm to a researcher or a study (Ojo, 2003). Population can be characterized as the aggregate of a specific people in a specific place. As indicated by Akinade and Owolabi (2009) population alludes to the aggregate arrangement of potential perceptions from which a specimen is drawn. It comprises of the total number of individuals of a specific kind that constitutes the question of enthusiasm for any study.

As opined by Ngechu (2006), a target population is a very much characterized set of individuals, administrations or components that are being researched. Since it was not pragmatic to include all the accessible population it was important to characterize an accessible population. Therefore the study population includes the academic staff of Covenant University, which has a population of 535 employees. These individuals provided information by answering the questionnaire on the status of motivation in the institution. The focus on the academic staff of the organization was based on the fact that academic staff of any university are the main crop of work force that drive performance.

**3.4 SAMPLE SIZE DETERMINATION**

Ojo (2003), opines that sampling is the way toward choosing agent components from a given population. It is likewise the components that can be chosen for research (Akinade & Owolabi Tude, 2009). Sampling design and sample size are very important to set up the representativeness of the sample for speculation (Berg, (2007). Along these lines, sampling is the subset of population or taking a bit of population with the end goal of summing up. For the purpose of this study the table for determining sample size was adopted. This is given below as:


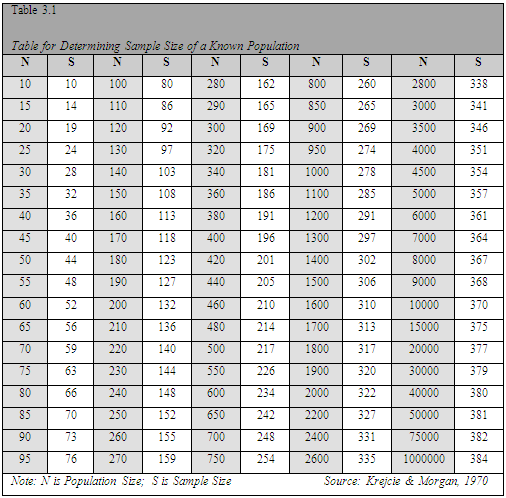


Source: Krejcie and Morgan 1970

**3.5 SAMPLING TECHNIQUES**

Simple random techniques are a standout amongst the most well-known sorts of irregular or probability sampling. In this technique, every individual from the population has an equivalent shot of being chosen as subject. The whole procedure of sampling is done in a solitary stride with each subject chose freely of alternate individuals from the population. This study adopted the simple random technique by giving individuals from the population copies of questionnaires at random in the work place.

**3.6 SAMPLE FRAME**

Bearden and Ingram (2007) expressed sampling frame as the synopsis or working depiction of the population utilized as a part of the sample determination. Sample frame can be characterized as the fundamental points of interest and particular of all individuals from a population from which a sample is to be drawn (Adebayo, 2000). The sample frame is the list or device used in identifying a population which helps to outline a set of elements which a target population can be derived from. It can be used to stand for all the elements of population of interest. In this study the sample frame is made up of the staff list of Covenant University sourced from the human resource department.

**3.7 SOURCE OF DATA COLLECTION**

As indicated by Owete (2014) data collection includes the way toward distinguishing and assembling information from important sources. Information for the survey was sourced from both the primary and secondary sources. Primary information regarding this research was information gathered from the field review led with the staff and management of Covenant University. .The primary source information for this study includes data collected through the distribution of copies of questionnaires to respondent of Covenant University employees. The secondary aspect needed to do with data sourced from books, articles, journals reports, covenant university library, internet and other important records which were profoundly identified with the topic understudy. This class of information had 95% of it utilized for the survey of related writing and the staying for defending the decision of specific choices taken.

**3.8 RESEARCH INSTRUMENTS AND DESIGN**

Ojo (2003) expressed that research instrument as apparatuses that are utilized to assemble data for the utilization of testing theories and offering answers to research questions. As indicated by Akinade and Owolabi (2009), questionnaire is the most broadly utilized in an overview consider, thus organized questionnaire, which comprises of close ended questions, was used. In this study, questionnaires are the significant instrument used. Questionnaire is viewed as a progression of inquiries, every one giving various option answers from which the respondents can pick (Punch, 2000). This is one of the normal instruments utilized as a part of social science research since they give a productive means by which factually quantifiable data can be gathered. The study likewise made use of copies of questionnaires on the grounds that a large number of the respondents were effectively reached and this additionally made data analysis extremely less difficult and simpler.

The items on the questionnaire were organized which were fundamentally closed ended type. In setting up the questionnaire, an introductory letter, directions and the primary body were set down. The questionnaires were controlled actually to the respondents. The other piece of the questionnaire is isolated into two. Segment A involves on inquiries in view of bio-data, which incorporates individual inquiries regarding the respondent, some of which are Gender and Age of the respondent. Segment B involves questions that are gone for endeavouring to give answers for the destinations of the study utilizing the 5-likert scale technique. The respondent would be made a request to connote or show whether they "Strongly Agree" (SA), "Agree" (An), "Undecided" (U), "Disagree" (D) or "Strongly Disagree" (SD) about the inquiries asked in this segment.

**3.9 RELIABILITY**

Internal consistency is a technique for reliability in which we judge how well the items on a test that are proposed to measure the same construct deliver similar results. A casual approach to test for internal consistency is just to contrast the answers with see in the event that they all concur with each other. In actuality, you will probably find a wide assortment of solutions, making it hard to see if internal consistency is great or not Andale (2016). A wide assortment of statistical tests is accessible for internal consistency; a standout amongst the most generally used is Cronbach's Alpha. Cronbach's alpha is the most well-known measure of internal consistency ("reliability"). It is most normally used when you have different Likert questions in a survey/questionnaire that shape a scale and you wish to decide whether the scale is reliable. T

The acceptable value from Cronbach alpha reliability analysis is that which ranges from 0.7-1.0.

| Reliability Statistics | |
| --- | --- |
| Cronbach's Alpha | N of Items |
| .747 | 23 |

Source: Field Survey, 2017.

The reliability statistics for this study is 0.747, therefore the instrument used for the study was adjudged.

**3.10 VALIDITY OF RESEARCH INSTRUMENT**

Borg and Gall (2009) characterize validity as how much a test measures what it indicates to measure. To guarantee validity, the researcher counselled the college supervisor who edit the questionnaire and exhorted on any essential changes. This guaranteed validity and reliability. Asikia (2006) expressed that validity can be characterized as the degree to which a measuring instrument measures what it is intended to measure. Content validity is not quite the same as face validity, which refers not to what the test really measures, but rather to what it externally seems to gauge. Face validity shows whether the test "looks legitimate" to the examinees who take it, the regulatory staff that choose its utilization and other in fact untrained onlookers. Content validity requires the utilization of perceived topic specialists to evaluate whether test things survey characterized content and more thorough statistical tests than does the appraisal of face validity. Content validity is regularly tended to in academic and vocational testing, where test things need to mirror the information really required for a given point range.

The face validity was done by giving staff of the academic unit of Covenant University to ascertain if the instrument captures the variables the researcher intended to measure. Content validity was carried out by presenting the questionnaire to my supervisor and other experts in the area to ensure it contained all the questions that is required to measure the variables before distribution.

**3.11 METHOD OF DATA COLLECTION AND PROCEDURE**

According to Blaxter (2005), primary data is characterized as comprising of materials that one has accumulated without anyone else's input through orderly perception, data chronicles, the result of questionnaires and interviews and contextual investigation which one has aggregated. Berg (2007), said that choosing a site or setting for a study it ought to be sensible in size and complexity so the study can be finished inside the time and spending plan accessible. This study collected both primary and secondary. Copies of questionnaire were used to collect primary data and journals, internet, and books were the source of secondary data collection.

**3.12 DATA ANALYSIS**

The data was analyzed utilizing statistical package for social sciences; (SPSS).The researcher referenced and coded all things on the questionnaire with the end goal of clear and succinct introduction of data. Quantitative data was produced by the study. To analyze numerical data, descriptive measurements information analysis technique was connected to analyze the numerical information utilizing shut finished inquiries. Descriptive statistical instruments, for example, diagrams, recurrence tables and percentages were utilized to demonstrate the aftereffects of the data. The descriptive statistics included the measure of focal propensity (weighted mean) and measures of dispersion. Plausible checks were directed and conflicting data was cleared fittingly. Statistical tests were run.

As indicated by Mugenda (2008), descriptive analyses are imperative since they give the establishment whereupon correlation and exploratory studies develop; they likewise give intimations with respect to the issues that ought to be centred on prompting to further studies. quantitative data were analyzed by means of Statistical Package for Social Sciences (SPSS) .The data were be cleaned, coded, arranged per each of the research variables. Linear regression was used to analyse the effect of the independent variables on the dependent variables. Linear regression was adopted in this study because the study is based on the effect of motivation on employee performance.

**CHAPTER FOUR**

**DATA ANALYSIS, FINDINGS AND DISCUSSION**

**4.0 Introduction**

This chapter contains the representation analysis and distribution of results in line with the study objectives. This chapter represents the descriptive statistics of items under the study. The statistical tools such as tables showing frequencies, percentages and mean were used to summaries findings from the survey. The presentation is guided by the research objectives and statistics were generated with the aim of generating responses for the research questions.

**4.1 Presentation of Data**

The responses generated from the questionnaire distributed among sampled respondents which are presented below. It is worth knowing that out of the 226 copies of questionnaire distributed to the respondents only 213 copies representing 94% of the total number of questionnaires distributed were successfully and returned.

**Table 4.1.1 Analysis of Response Rate**

| **Questionnaire** | **Respondent** | **Percentage of response** |
| --- | --- | --- |
| Returned | 213 | 94% |
| Not returned | 13 | 6% |
| **Total Distributed** | 226 | 100% |

**Sources: Field Survey, 2017.**

**4.2 Data Analysis and Interpretations**

This sub section below shows the data collected from the responses of the copies of questionnaires.it includes the demographic characteristics of respondents.

**Table 4.2.1**

| **Gender of respondents** | | | | | |
| --- | --- | --- | --- | --- | --- |
|  | | Frequency | Percent | Valid Percent | Cumulative Percent |
| Valid | Male | 100 | 46.9 | 46.9 | 46.9 |
|  | Female | 113 | 53.1 | 53.1 | 100.0 |
|  | Total | 213 | 100.0 | 100.0 |  |

**Source: Field Survey, 2017**

Table 4.2.1 shows the frequency distribution by gender of respondents. Where 100 (46.9%) of the respondents are male and 113 (53.1%) of the respondents are female. Showing the views from the respondents were from both genders. Majority of the respondents were female.

**Table 4.2.2**

| **Age of respondent** | | | | | |
| --- | --- | --- | --- | --- | --- |
|  | | Frequency | Percent | Valid Percent | Cumulative Percent |
| Valid | 21-30 | 80 | 37.6 | 37.6 | 37.6 |
|  | 31-40 | 95 | 44.6 | 44.6 | 82.2 |
|  | 41-50 | 26 | 12.2 | 12.2 | 94.4 |
|  | 61 and above | 12 | 5.6 | 5.6 | 100.0 |
|  | Total | 213 | 100.0 | 100.0 |  |

. **Sources: Field Survey, 2017**

Table 4.2.2 shows the age of the respondent. The table shows that 80 (37.6%) of the respondents fall under the ages of 21-30 years, 95 (44.6%) respondent are between the ages of 31-40 years,26 (12.2%) respondents are within the ages of 41-50, while 12 (5.6%) respondents are 61 and above years respectively. Majority of the respondents were between the ages of 31-40 years of age.

**Table 4.2.3**

| **Marital Status of respondents** | | | | | |
| --- | --- | --- | --- | --- | --- |
|  | | Frequency | Percent | Valid Percent | Cumulative Percent |
| Valid | Single | 82 | 38.5 | 38.5 | 38.5 |
|  | Married | 107 | 50.2 | 50.2 | 88.7 |
|  | Divorced | 4 | 1.9 | 1.9 | 90.6 |
|  | Widowed | 16 | 7.5 | 7.5 | 98.1 |
|  | Separated | 4 | 1.9 | 1.9 | 100.0 |
|  | Total | 213 | 100.0 | 100.0 |  |

**Sources: Field Survey, 2017.**

Table 4.2.3 presents the marital status of the respondents. It shows that 82 (38.5%) of the respondents are single, 107 (50.2%) of the respondent are married, 4 (1.9%) of the respondents are divorced, 16 (7.5%) of the respondents are widowed and 4 (1.9%) of the respondent are separated. Majority of the respondents are married.

**Table 4.2.4**

| **Highest Academic Qualification of respondents** | | | | | |
| --- | --- | --- | --- | --- | --- |
|  | | Frequency | Percent | Valid Percent | Cumulative Percent |
| Valid | B.Sc./HND | 34 | 16.0 | 16.0 | 16.0 |
|  | M.Sc./MBA | 106 | 49.8 | 49.8 | 65.7 |
|  | PHD | 73 | 34.3 | 34.3 | 100.0 |
|  | Total | 213 | 100.0 | 100.0 |  |

. **Sources: Field Survey, 2017**

Table 4.2.4 depicts the highest educational qualification of the respondents. The table presents that 34 (16%) of the respondents were holders of B.Sc./HND, 106 (49.8%) of the respondents are holders of M.Sc./MBA and 73 (34.3%) of the respondents are holders of PHD. Therefore it can be deduced that majority of the respondents were holders of the M.Sc./MBA educational qualification.

**Table 4.2.5**

| **Number of years worked** | | | | | |
| --- | --- | --- | --- | --- | --- |
|  | | Frequency | Percent | Valid Percent | Cumulative Percent |
| Valid | 0-3 | 80 | 37.6 | 37.6 | 37.6 |
|  | 4-6 | 80 | 37.6 | 37.6 | 75.1 |
|  | 7-9 | 28 | 13.1 | 13.1 | 88.3 |
|  | 10 and above | 19 | 8.9 | 8.9 | 97.2 |
|  | 5 | 6 | 2.8 | 2.8 | 100.0 |
|  | Total | 213 | 100.0 | 100.0 |  |

**Source: Field of Survey. 2017.**

Table 4.2.5 presents the number of years worked in the institution. The table shows that 80 (37.6%) respondents worked between 0-3 years, 80 (37.6%) respondents worked between 4-6 years, 28 (13.1%) respondents worked between 7-9 years and 19 (8.9%) respondents worked between 10 and above years.

**Table 4.2.6**

| **The compensation scheme is very favourable and motivates me to effectively carry out my duties** | | | | | |
| --- | --- | --- | --- | --- | --- |
|  | | Frequency | Percent | Valid Percent | Cumulative Percent |
| Valid | SD | 4 | 1.9 | 1.9 | 1.9 |
|  | D | 33 | 15.5 | 15.5 | 17.4 |
|  | U | 36 | 16.9 | 16.9 | 34.3 |
|  | A | 136 | 63.8 | 63.8 | 98.1 |
|  | SA | 4 | 1.9 | 1.9 | 100.0 |
|  | Total | 213 | 100.0 | 100.0 |  |

**Source: Field Survey, 2017.**

Table 4.2.6 shows that 4 (1.9%) of the respondents strongly disagree that the compensation scheme is very favourable and motivates me to effectively carry out my duties, 33 (15.5%) of respondents disagree, 36(16.9%) of the respondents are undecided, 136 (63.8%) of the respondents agree and 4 (1.9%) of the respondents strongly agree. Therefore it can be deduced that majority of the respondents agree that the compensation scheme is very favourable and motivates me to effectively carry out my duties.

**Table 4.2.7**

| **The compensation package enhances my commitment to the institutional goals** | | | | | |
| --- | --- | --- | --- | --- | --- |
|  | | Frequency | Percent | Valid Percent | Cumulative Percent |
| Valid | SD | 16 | 7.5 | 7.5 | 7.5 |
|  | D | 38 | 17.8 | 17.8 | 25.4 |
|  | U | 31 | 14.6 | 14.6 | 39.9 |
|  | A | 128 | 60.1 | 60.1 | 100.0 |
|  | Total | 213 | 100.0 | 100.0 |  |

**Source: Field Survey, 2017.**

Table 4.2.7 shows that 16 (7.5%) of the respondents strongly disagree that the compensation package enhances my commitment to the institutional goals, 38 (17.8%) of the respondents disagree, 31 (14.6%) of the respondents were undecided, 128 (60.1%) of the respondents agree. Therefore, it can be deduced that majority of the respondents agree that the compensation package enhances my commitment to the institutional goals.

**Table 4.2.8**

| **I am motivated because the compensation scheme is both monetary and non-monetary** | | | | | |
| --- | --- | --- | --- | --- | --- |
|  | | Frequency | Percent | Valid Percent | Cumulative Percent |
| Valid | SD | 16 | 7.5 | 7.5 | 7.5 |
|  | D | 28 | 13.1 | 13.1 | 20.7 |
|  | U | 41 | 19.2 | 19.2 | 39.9 |
|  | A | 122 | 57.3 | 57.3 | 97.2 |
|  | SA | 6 | 2.8 | 2.8 | 100.0 |
|  | Total | 213 | 100.0 | 100.0 |  |

**Source: Field Survey, 2017**.

Table 4.2.9 shows that 16 (7.5%) of respondents strongly disagree that I am motivated because of the compensation scheme is both monetary and non-monetary, 28 (13.1%) of the respondents disagree, 41 (19.2%) of the respondents are undecided, 122 (57.3%) of the respondents agree and 6 (2.8%) of the respondents strongly agree. Therefore it can be deduced that majority of the respondents agree that they are motivated because the compensation scheme is both monetary and non-monetary.

**Table 4.2.10**

| **Training and development programs are encouraged in this institution for capacity building** | | | | | |
| --- | --- | --- | --- | --- | --- |
|  | | Frequency | Percent | Valid Percent | Cumulative Percent |
| Valid | D | 10 | 4.7 | 4.7 | 4.7 |
|  | U | 4 | 1.9 | 1.9 | 6.6 |
|  | A | 167 | 78.4 | 78.4 | 85.0 |
|  | SA | 32 | 15.0 | 15.0 | 100.0 |
|  | Total | 213 | 100.0 | 100.0 |  |

**Source: Field Survey, 2017.**

Table 4.2.10 shows that 10 (4.7%) of the respondents disagree that training and development programs are encouraged in this institution for capacity building, 4 (1.9%) of the respondents were undecided, 167 (78.4%) of the respondents agree and 32 (15%) of the respondents strongly agree. Therefore it can be deduced that majority of the respondents agree that training and development programs are encouraged in the institution for capacity building.

**Table 4.2.11**

| **Training and development regime in this institution enhances my career development** | | | | | |
| --- | --- | --- | --- | --- | --- |
|  | | Frequency | Percent | Valid Percent | Cumulative Percent |
| Valid | SD | 16 | 7.5 | 7.5 | 7.5 |
|  | U | 20 | 9.4 | 9.4 | 16.9 |
|  | A | 167 | 78.4 | 78.4 | 95.3 |
|  | SA | 10 | 4.7 | 4.7 | 100.0 |
|  | Total | 213 | 100.0 | 100.0 |  |

**Source: Field Survey, 2017.**

Table 4.2.11 presents that 16 (7.5%) of the respondents strongly disagree that training and development regime in this institution enhances my career development, 20 (9.4%) of respondents were undecided, 167 (78.4%) of respondents agree and 10 (4.7%) of respondents strongly agree. Therefore I deduced that majority of respondents agree that the training and development regime in this institution enhances my career development.

**Table 4.2.12**

| **Training and development programs in this institution facilitate creativity in the workplace** | | | | | |
| --- | --- | --- | --- | --- | --- |
|  | | Frequency | Percent | Valid Percent | Cumulative Percent |
| Valid | D | 4 | 1.9 | 1.9 | 1.9 |
|  | U | 20 | 9.4 | 9.4 | 11.3 |
|  | A | 165 | 77.5 | 77.5 | 88.7 |
|  | SA | 24 | 11.3 | 11.3 | 100.0 |
|  | Total | 213 | 100.0 | 100.0 |  |

**Source: Field Survey, 2017.**

Table 4.2.12 shows that 4 (1.9%) of respondents disagree that training and development programs in this institution facilitate creativity in the workplace, 20 (9.4%) of the respondents were undecided, 165 (77.5%) of respondents agree and 24 (11.3%) of respondents strongly agree. Therefore I deduced that majority of the respondents agree that training and development programs in this institution facilitate creativity in the workplace.

**Table 4.2.13**

| **The institution allows flexible working hours** | | | | | |
| --- | --- | --- | --- | --- | --- |
|  | | Frequency | Percent | Valid Percent | Cumulative Percent |
| Valid | SD | 32 | 15.0 | 15.0 | 15.0 |
|  | D | 78 | 36.6 | 36.6 | 51.6 |
|  | U | 40 | 18.8 | 18.8 | 70.4 |
|  | A | 59 | 27.7 | 27.7 | 98.1 |
|  | SA | 4 | 1.9 | 1.9 | 100.0 |
|  | Total | 213 | 100.0 | 100.0 |  |

**Source: Field Survey, 2017.**

Table 4.2.13 shows that 32 (15%) of the respondents strongly disagree, 73 (36.6%) of the respondents disagree, 40 (18.8%) of the respondents were undecided, 59 (27.7%) of the respondents agree and 4 (1.9%) of the respondents strongly agree. Therefore I deduced that majority of the respondents disagree that the institution allows flexible working hours.

**Table 4.2.14**

| **The institution makes provision of both maternity and paternity leave** | | | | | |
| --- | --- | --- | --- | --- | --- |
|  | | Frequency | Percent | Valid Percent | Cumulative Percent |
| Valid | SD | 30 | 14.1 | 14.1 | 14.1 |
|  | D | 78 | 36.6 | 36.6 | 50.7 |
|  | U | 29 | 13.6 | 13.6 | 64.3 |
|  | A | 72 | 33.8 | 33.8 | 98.1 |
|  | SA | 4 | 1.9 | 1.9 | 100.0 |
|  | Total | 213 | 100.0 | 100.0 |  |

**Source: Field Survey, 2017.**

Table 4.2.14 shows that 30 (14.1%) of the respondents strongly disagree that the institution makes provision of both maternity and paternity leave, 78 (36.6%) of the respondents disagree, 29 (13.6%) of the respondents were undecided, 72 (33.8%) of the respondents agree and 4 (1.9%) of the respondents strongly agree. Therefore I deduced that majority of the respondents disagree that the institution makes provision of both maternity and paternity leave.

**Table 4.2.15**

| **The flexible working hours enhances my productivity** | | | | | |
| --- | --- | --- | --- | --- | --- |
|  | | Frequency | Percent | Valid Percent | Cumulative Percent |
| Valid | SD | 20 | 9.4 | 9.4 | 9.4 |
|  | D | 66 | 31.0 | 31.0 | 40.4 |
|  | U | 46 | 21.6 | 21.6 | 62.0 |
|  | A | 63 | 29.6 | 29.6 | 91.5 |
|  | SA | 18 | 8.5 | 8.5 | 100.0 |
|  | Total | 213 | 100.0 | 100.0 |  |

**Source: Field Survey, 2017.**

Table 4.2.15 shows that 20 (9.4%) of the respondents strongly disagree that the flexible working hours enhances my productivity, 66 (31.0%) of the respondents disagree, 46 (21.6%) of the respondents were undecided, 63 (29.6%) of the respondents agree and 18 (8.5%) of the respondents strongly agree. Therefore I deduced that majority of the respondents disagree that the flexible working hours enhances my productivity.

**Table 4.2.16**

| **I am able to balance my work and life and have excellent result** | | | | | |
| --- | --- | --- | --- | --- | --- |
|  | | Frequency | Percent | Valid Percent | Cumulative Percent |
| Valid | D | 36 | 16.9 | 16.9 | 16.9 |
|  | U | 22 | 10.3 | 10.3 | 27.2 |
|  | A | 147 | 69.0 | 69.0 | 96.2 |
|  | SA | 8 | 3.8 | 3.8 | 100.0 |
|  | Total | 213 | 100.0 | 100.0 |  |

**Source: Field Survey. 2017**.

Table 4.2.16 shows that 36 (16.9%) of the respondents disagree that I am able to balance my work and life and have excellent result, 22 (10.3%) of respondents were undecided, 147 (69%) of respondents agree, 8 (3.8%) of respondents strongly agree. Therefore I deduced that majority of the respondents agree that I am able to balance my work life and have excellent result.

**Table 4.2.17**

| **There are adequate facilities that foster my productivity** | | | | | |
| --- | --- | --- | --- | --- | --- |
|  | | Frequency | Percent | Valid Percent | Cumulative Percent |
| Valid | D | 10 | 4.7 | 4.7 | 4.7 |
|  | U | 35 | 16.4 | 16.4 | 21.1 |
|  | A | 160 | 75.1 | 75.1 | 96.2 |
|  | SA | 8 | 3.8 | 3.8 | 100.0 |
|  | Total | 213 | 100.0 | 100.0 |  |

**Source: Field Survey, 2017**.

Table 4.2.17 shows that 10 (4.7%) of the respondents disagree that there are adequate facilities that foster my productivity, 35 (16.4%) of the respondents were undecided, 160 (75.1%) of the respondents agree and 8 (3.8%) of the respondents strongly agree. Therefore I deduced that majority of the respondents agree that there are adequate facilities that foster my productivity.

**Table 4.2.18**

| **My productivity depends on how i am motivated** | | | | | |
| --- | --- | --- | --- | --- | --- |
|  | | Frequency | Percent | Valid Percent | Cumulative Percent |
| Valid | SD | 4 | 1.9 | 1.9 | 1.9 |
|  | D | 22 | 10.3 | 10.3 | 12.2 |
|  | U | 35 | 16.4 | 16.4 | 28.6 |
|  | A | 132 | 62.0 | 62.0 | 90.6 |
|  | SA | 20 | 9.4 | 9.4 | 100.0 |
|  | Total | 213 | 100.0 | 100.0 |  |

**Source: Field Survey, 2017.**

Table 4.2.18 shows that 4 (1.9%) of the respondents strongly disagree that my productivity depends on how I am motivated, 22 (10.3%) of the respondents disagree, 35 (16.4%) of the respondents were undecided, 132 (62%) of the respondents agree and 20 (9.4%) of the respondents strongly agree. Therefore I deduced that majority of the respondents agree that my productivity depends on how I am motivated.

**Table 4.2.19**

| **Adequate facilities are provided to promote innovation** | | | | | |
| --- | --- | --- | --- | --- | --- |
|  | | Frequency | Percent | Valid Percent | Cumulative Percent |
| Valid | SD | 4 | 1.9 | 1.9 | 1.9 |
|  | D | 10 | 4.7 | 4.7 | 6.6 |
|  | U | 46 | 21.6 | 21.6 | 28.2 |
|  | A | 137 | 64.3 | 64.3 | 92.5 |
|  | SA | 16 | 7.5 | 7.5 | 100.0 |
|  | Total | 213 | 100.0 | 100.0 |  |

**Source: Field Survey, 2017**.

Table 4.2.19 shows that 4 (1.9%) of the respondents strongly disagree that adequate facilities are provided to promote innovation, 10 (4.7%) of the respondents disagree, 46 (21.6%) of the respondents were undecided, 137 (64.3%) of the respondents agree and 16 (7.5%) of the respondents strongly agree. Therefore I deduced that majority of the respondents agree that adequate facilities are provided to promote innovation.

**Table 4.2.20**

| **Team work promotes innovation in the institution** | | | | | |
| --- | --- | --- | --- | --- | --- |
|  | | Frequency | Percent | Valid Percent | Cumulative Percent |
| Valid | SD | 4 | 1.9 | 1.9 | 1.9 |
|  | D | 16 | 7.5 | 7.5 | 9.4 |
|  | U | 24 | 11.3 | 11.3 | 20.7 |
|  | A | 153 | 71.8 | 71.8 | 92.5 |
|  | SA | 16 | 7.5 | 7.5 | 100.0 |
|  | Total | 213 | 100.0 | 100.0 |  |

**Source: Field Survey, 2017.**

Table 4.2.20 shows that 4 (1.9%) of the respondents strongly disagree that team work promotes innovation in the institution, 16 (7.5%) of the respondents disagree, 24 (11.3%) of the respondents were undecided, 153 (71.8%) of the respondents agree and 16 (7.5%) of the respondents strongly agree.

**Table 4.2.21**

| **The working condition enables me to be innovative** | | | | | |
| --- | --- | --- | --- | --- | --- |
|  | | Frequency | Percent | Valid Percent | Cumulative Percent |
| Valid | SD | 10 | 4.7 | 4.7 | 4.7 |
|  | D | 6 | 2.8 | 2.8 | 7.5 |
|  | U | 44 | 20.7 | 20.7 | 28.2 |
|  | A | 153 | 71.8 | 71.8 | 100.0 |
|  | Total | 213 | 100.0 | 100.0 |  |

**Source: Field Survey, 2017.**

Table 4.2.21 shows that 10 (4.7%) of respondents strongly disagree that the working condition enables me to be innovative, 6 (2.8%) of the respondents disagree, 44 (20.7%) of respondents were undecided and 153 (71.8%) agree. Therefore I deduced that majority of the respondents agree that the working condition enables me to be innovative.

**Table 4.2.22**

| **I am dedicated towards the achievement of the goals and objectives of the institution** | | | | | |
| --- | --- | --- | --- | --- | --- |
|  | | Frequency | Percent | Valid Percent | Cumulative Percent |
| Valid | SD | 4 | 1.9 | 1.9 | 1.9 |
|  | U | 12 | 5.6 | 5.6 | 7.5 |
|  | A | 151 | 70.9 | 70.9 | 78.4 |
|  | SA | 46 | 21.6 | 21.6 | 100.0 |
|  | Total | 213 | 100.0 | 100.0 |  |

**Source: Field Survey, 2017.**

Table 4.2.22 shows that 4 (1.9%) of the respondents strongly disagree that I am dedicated towards the achievement of the goals and objectives of the institution, 12 (5.6%) of the respondents were undecided, 151 (70.9%) of the respondents agree and 46 (21.6%) of the respondents strongly agree. Therefore I deduced that majority of the respondents agree that I am dedicated towards the achievement of the goals and objectives of the institution.

**Table 4.2.23**

| **I am loyal to this institution because i am highly motivated** | | | | | |
| --- | --- | --- | --- | --- | --- |
|  | | Frequency | Percent | Valid Percent | Cumulative Percent |
| Valid | SD | 4 | 1.9 | 1.9 | 1.9 |
|  | D | 12 | 5.6 | 5.6 | 7.5 |
|  | U | 56 | 26.3 | 26.3 | 33.8 |
|  | A | 125 | 58.7 | 58.7 | 92.5 |
|  | SA | 16 | 7.5 | 7.5 | 100.0 |
|  | Total | 213 | 100.0 | 100.0 |  |

**Source: Field Survey, 2017.**

Table 4.2.23 shows that 4 (1.9%) of the respondents strongly disagree that I am loyal to this institution because I am highly motivated, 12 (5.6%) of the respondents disagree, 56 (26.3%) of the respondents were undecided, 125 (58.7%) of the respondents agree and 16 (7.5%) of the respondents strongly agree. Therefore I deduced that majority of the respondents agree that I am loyal to this institution because I am highly motivated.

**Table 4.2.24**

| **I feel it is 'morally correct' to dedicate myself to this institution** | | | | | |
| --- | --- | --- | --- | --- | --- |
|  | | Frequency | Percent | Valid Percent | Cumulative Percent |
| Valid | SD | 4 | 1.9 | 1.9 | 1.9 |
|  | D | 10 | 4.7 | 4.7 | 6.6 |
|  | U | 28 | 13.1 | 13.1 | 19.7 |
|  | A | 145 | 68.1 | 68.1 | 87.8 |
|  | SA | 26 | 12.2 | 12.2 | 100.0 |
|  | Total | 213 | 100.0 | 100.0 |  |

**Source: Field Survey, 2017**.

Table 4.2.24 shows that 4 (1.9%) of the respondents strongly disagree that I feel it is ‘morally correct’ to dedicate myself to this institution, 10 (4.7%) of the respondents disagree. 28 (13.1%) of the respondents were undecided, 145 (68.1%) of the respondents agree and 26 (12.2%) of respondents strongly agree. Therefore I deduced that majority of the respondent agree that I feel it is ‘morally correct’ to dedicate myself to this institution.

**4.3** **Hypotheses Testing and Discussion of Results**

**Test of Hypothesis One**

Objective 1: To determine the effect of compensation on employee commitment

Research question: To what degree is the effect of compensation on employee commitment?

**H_01:_** Compensation does not have effect on employee commitment.

**H _a1:_** Compensation has effect on employee’s commitment.

The researcher made use of the Regression (ANOVA) analysis to test this. The analysis is presented below: -

**Table 4.3.1**

| **Model Summary** | | | | |
| --- | --- | --- | --- | --- |
| Model | R | R Square | Adjusted R Square | Std. Error of the Estimate |
| 1 | .438^a^ | .192 | .188 | .513 |
| a. Predictors: (Constant), COMPEN | | | | |

**Source: Field Survey, 2017.**

**Decision Rule**: Reject the null hypothesis when the level of significance falls below 0.05 and accept the null hypothesis when the level of significance is above 0.05.

**Interpretation**: table 4.3.1 the model summary table, shows that R square which represents the effect of compensation on employee commitment is 0.192. This denotes a moderate relationship between compensation on employee commitment. Also, the table explains the degree of variance of the dependent variable (employee commitment) that is described by the independent variable (compensation). The value of R squared in the model summary table above is 0.192 and 19.2% in percentage. This therefore connotes that our model explains 19.2% variance of the dependent variable.

**Decision**: compensation has a moderate influence on employee commitment.

**Discussion of result**: compensation contributes to 19.2% of employee commitment in the institution. The regression effect of the independent variable (compensation) on the dependent variable employee commitment) was 0.438, which is characterized as moderate.

**Table 4.3.2**

| **ANOVA^a^** | | | | | | |
| --- | --- | --- | --- | --- | --- | --- |
| Model | | Sum of Squares | Df | Mean Square | F | Sig. |
| 1 | Regression | 13.229 | 1 | 13.229 | 50.186 | .000^b^ |
|  | Residual | 55.620 | 211 | .264 |  |  |
|  | Total | 68.849 | 212 |  |  |  |
| a. Dependent Variable: COMM | | | | | | |
| b. Predictors: (Constant), COMPEN  **Source: Field Survey, 2017**. | | | | | | |

The objective of Table 4.3.2 is to show the statistical significance of the result obtained. The aim of the ANOVA table is to test whether the null hypothesis is statistically significant. The results from the table denote that the model is statistically significant with a significance level at 0.00, with an F-value of 50.186.

**Discussion**: Hence, we would reject the null hypothesis following the decision rule earlier stated.

**Discussion of Result**: The results from the table show that there is a significant influence of compensation on employee commitment.

| **Table 4.3.3**  **Coefficients** | | | | | | |
| --- | --- | --- | --- | --- | --- | --- |
| Model | | Unstandardized Coefficients | | Standardized Coefficients | T | Sig. |
|  |  | B | Std. Error | Beta |  |  |
| 1 | (Constant) | 2.858 | .146 |  | 19.556 | .000 |
|  | COMPEN | .298 | .042 | .438 | 7.084 | .000 |
| a. Dependent Variable: COMM | | | | | | |

**Source: Field Survey, 2017**.

**Interpretation of result**: Table 4.3.3 has the B unstandardized co-efficient at 0.298, which reflects a direct influence of compensation on employee commitment. This connotes that a unit increase in compensation will cause a 0.298 increase on employee commitment. This influence also has a significant level at 0.00.

**Decision**: This indicates a significant direct influence of compensation on employee commitment with a level of significance at 0.00, which is less than 0.05.

**Discussion of Result**: The B coefficient of compensation (independent variable) which is 0.298, indicates a direct influence of it on employee commitment (dependent variable).

**Testing of Hypothesis Two**

Objective 2: To measure the extent to which training and development affects employee’s innovation/creativity.

Research Question 2: To what extent does training and development affect employee’s innovation/creativity?

**H_02:_** Training and development does not affect employee’s creativity.

**H_a2:_** Training and development affects employee’s creativity.

The researcher made use of the Regression (ANOVA) analysis to test this. The analysis is presented below: -

**Table 4.3.4**

| **Model Summary** | | | | |
| --- | --- | --- | --- | --- |
| Model | R | R Square | Adjusted R Square | Std. Error of the Estimate |
| 1 | .550^a^ | .303 | .300 | .483 |
| a. Predictors: (Constant), TD | | | | |

**Source: Field Survey, 2017**.

**Decision Rule:** “R square” in the table above portrays the strength of the existing relationship between both variables, therefore, when R square is between 0.0 and 0.20, this shows there is a weak relationship; 0.20 to 0.40 denotes a medium relationship; 0.40 to 0.60 depicts a moderate relationship; 0.60 to 0.80 denotes a strong relationship, while greater than 0.80 denotes a very strong relationship. Reject the null hypothesis when the level of significance falls below 0.05 and accept the null hypothesis when the level of significance is above 0.05.

**Interpretation:** Table 4.3.4 the model summary table, shows that R square which represents the extent to which training and development affects employee’s innovation/creativity is 0.303. This denotes a medium relationship between training and development on employee innovation. Also, the table explains the degree of variance of the dependent variable (employee innovation) that is described by the independent variable (training and development). The value of R squared in the model summary table above is 0.303 and 30.3% in percentage. This therefore connotes that our model explains 30.3% variance of the dependent variable.

**Decision:** training and development has a medium influence on employee innovation.

**Discussion of result:** Labeling contributes to 30.3% of consumer’s information search. The regression effect of the independent variable (training and development) on the dependent variable (employee innovation) was 0.550, which is characterized as moderate.

**Table 4.3.5**

| **ANOVA^a^** | | | | | | |
| --- | --- | --- | --- | --- | --- | --- |
| Model | | Sum of Squares | Df | Mean Square | F | Sig. |
| 1 | Regression | 21.421 | 1 | 21.421 | 91.737 | .000^b^ |
|  | Residual | 49.269 | 211 | .234 |  |  |
|  | Total | 70.690 | 212 |  |  |  |
| a. Dependent Variable: INNO | | | | | | |
| b. Predictors: (Constant), TD | | | | | | |

**Source: Field Survey, 2017.**

**Interpretation:** the objective of Table 4.3.4 is to show the statistical significance of the result obtained. The aim of the ANOVA table is to test whether the null hypothesis is statistically significant. The results from the table denote that the model is statistically significant with a significance level at 0.00, with an F-value of 91.737.

**Decision:** Hence, we would reject the null hypothesis following the decision rule earlier stated.

**Discussion of Result:** The results from the table show that there is a significant influence of training and development on employee innovation.

**Table 4.3.5**

| **Coefficients** | | | | | | |
| --- | --- | --- | --- | --- | --- | --- |
| Model | | Unstandardized Coefficients | | Standardized Coefficients | T | Sig. |
|  |  | B | Std. Error | Beta |  |  |
| 1 | (Constant) | 1.131 | .269 |  | 4.204 | .000 |
|  | TD | .653 | .068 | .550 | 9.578 | .000 |
| a. Dependent Variable: INNO | | | | | | |

**Source: Field Survey, 2017.**

**Interpretation of Result:** Table 4.3.5 has the B unstandardized co-efficient at 0.653, which represents the extent to which training and development affects employee innovation. This connotes that a unit increase in training and development will cause a 0.653 increase on employee innovation. This influence also has a significant level at 0.00.

**Decision:** This indicates a significant direct influence of training and development on employee innovation with a level of significance at 0.00, which is less than 0.05.

**Discussion of Result:** The B coefficient of training and development (independent variable) which is 0.653 indicates a direct influence of it on employee innovation (dependent variable).

**Testing of Hypothesis Three**

Objective 3: To determine the role of work life balance on employee’s productivity

Research Question 3: In what ways does work life balance impact employee’s productivity?

**H_03:_** Work life balance does not impact employee’s productivity.

**H_a3:_** Work life balance has an impact on employee productivity.

The researcher made use of the Regression (ANOVA) analysis to test this. The analysis is presented below: -

**Table 4.3.6**

| **Model Summary** | | | | |
| --- | --- | --- | --- | --- |
| Model | R | R Square | Adjusted R Square | Std. Error of the Estimate |
| 1 | .241^a^ | .058 | .053 | .485 |
| a. Predictors: (Constant), WLB | | | | |

**Source: Field Survey, 2017.**

**Decision Rule:** “R square” in the table above portrays the strength of the existing relationship between both variables, therefore, when R square is between 0.0 and 0.20, this shows there is a weak relationship; 0.20 to 0.40 denotes a medium relationship; 0.40 to 0.60 depicts a moderate relationship; 0.60 to 0.80 denotes a strong relationship, while greater than 0.80 denotes a very strong relationship. Reject the null hypothesis when the level of significance falls below 0.05 and accept the null hypothesis when the level of significance is above 0.05.

**Interpretation:** Table 4.3.6, the model summary table, shows that R square which represents the role of work life balance on employee’s productivity value is 0.058. This denotes a weak relationship between work life balances on employee’s productivity. Also, the table explains the degree of variance of the dependent variable (employee productivity) that is described by the independent variable (work life balance). The value of R squared in the model summary table above is 0.058 and 5.8% in percentage. This therefore connotes that our model explains 5.8% variance of the dependent variable.

**Decision:** work life balance has a weak influence on employee productivity.

**Discussion of result:** work life balance contributes to 5.8% of employee productivity. The regression effect of the independent variable (work life balance) on the dependent variable (employee productivity) was 0.241, which is characterized as weak.

**Table 4.3.7**

| **ANOVA^a^** | | | | | | |
| --- | --- | --- | --- | --- | --- | --- |
| Model | | Sum of Squares | Df | Mean Square | F | Sig. |
| 1 | Regression | 3.054 | 1 | 3.054 | 12.969 | .000^b^ |
|  | Residual | 49.682 | 211 | .235 |  |  |
|  | Total | 52.736 | 212 |  |  |  |
| a. Dependent Variable: PRO | | | | | | |
| b. Predictors: (Constant), WLB | | | | | | |

**Source: Field Survey, 2017.**

**Interpretation:** the objective of Table 4.3.7 is to show the statistical significance of the result obtained. The aim of the ANOVA table is to test whether the null hypothesis is statistically significant. The results from the table denote that the model is statistically significant with a significance level at 0.00, with an F-value of 12.969.

**Decision:** Hence, we would reject the null hypothesis following the decision rule earlier stated.

**Discussion of Result:** The results from the table show that there is a significant influence of work life balance on employee productivity.

**Table 4.3.8**

| **Coefficients** | | | | | | |
| --- | --- | --- | --- | --- | --- | --- |
| Model | | Unstandardized Coefficients | | Standardized Coefficients | T | Sig. |
|  |  | B | Std. Error | Beta |  |  |
| 1 | (Constant) | 3.320 | .106 |  | 31.463 | .000 |
|  | WLB | .130 | .036 | .241 | 3.601 | .000 |
| a. Dependent Variable: PRO | | | | | | |

**Source: Field Survey, 2017.**

**Interpretation of Result:** Table 4.3.8 has the B unstandardized co-efficient at 0.130, which reflects a direct influence of work life balance on employee productivity. This connotes that a unit increase in work life balance will cause a 0.130 increase in employee productivity. This influence also has a significant level at 0.000.

**Decision:** This indicates a significant direct influence of work life balance on employee productivity with a level of significance at 0.000, which is less than 0.05.

**Discussion of Result:** The B coefficient of work life balance (independent variable) which is 0.130, indicates a direct influence of it on employee productivity (dependent variable).

**Discussion of Findings:** Findings from Hypothesis one shows that Compensation has effect on employee commitment. This is in line with the study of Whitener (2004) who looked at compensation packages and its effect on employee commitment.

Results from Hypothesis two suggest that training and development affects employee creativity/innovation. This confirms the study of Salman (2014), who examined the impact of training and development on employee creativity.

Hypothesis three shows that Work life balance impacts on employee productivity. This aligns with the study of Chimani (2015), who assessed the impact of work-life balance on employee productivity.

**Chapter Five**

**Summary, Conclusion and Recommendations**

**5.0 Introduction**

The aim of this study is to investigate the effect of motivation on employee performance using Covenant University academic staff as a case study. This chapter however, discusses both the theoretical and empirical findings of the study. The results of this study presented were disclosed. In the course of this research work, the hypotheses stated for the study guided the arrangement of the discussion. The result of the test proves that motivation can be used as a strategy for enhancing employee performance and all undertaken options should be implemented followed by the conclusions drawn from the findings. Recommendations and suggestions for further reading were included.

**5.1 Summary of the work**

This research work has been conducted based on the current study titled “Effect of motivation on employee performance” was fully introduced with reference to the background of the study, the problem of the study, research objectives and other relevant areas in understanding the topic. The second chapter reviewed literature relevant to the study looking at the works of other researchers with respect of motivation, theories that underpin both motivation and employee performance and other related publications. Chapter three explains the research method adopted. Adopting the descriptive research method and using table for determining sample size the researcher arrived at 226 respondents for the study and obtained data from them via well administered questionnaires. The data retrieved was later analyzed and interpreted in the fourth chapter of the study using the mathematical tool, Statistical Product and Service Solutions (SPSS) version 20.

Finally, chapter five included a general summary of the entire research work, which consisted of summary findings, conclusion of the study, and suggested recommendations for the implementations of motivation to other institution.

**5.2 Research Findings**

The findings of this study are divided into two (2) namely; theoretical findings and empirical findings. The theoretical findings are drawn from the literature review in chapter two (2) while the empirical findings are derived from the data generated from the field survey.

**5.2.1 Summary of Theoretical Findings**

Revealed the following findings:

1. This theory was propounded by Abraham Maslow. It depended on the suspicion that employees are inspired by arrangement of five widespread needs, this needs include self-actualization, esteem needs, belongingness and love needs, safety needs and biological and physiological needs. These scope of needs he asserted the individual will be persuaded to satisfy whichever is most capable at the desperate hour Maslow (1970). Maslow (1943) expressed that individuals are motivated to accomplish certain needs and that a few needs outweigh others.
2. According to Victor Vroom a therapist in 1964, he propounded the expectancy theory. The theory is just pertinent to a work setting that depends on people’s expectations. Vroom through these standards looked to depict that motivation is a component of the relationship between; effort exhausted by an individual and saw level of performance; and the desire that reward for coveted results will be identified with performance. The expectancy theory in view of these assumptions has three key components: expectancy, instrumentality, and valence. An individual is motivated to the extent that he or she trusts that (an) effort will prompt to adequate performance (expectancy), (b) performance will be compensated (instrumentality), and (c) the value of the reward is exceedingly positive (valence).
3. The reactance theory, this psychological theory depicts how individuals respond when they sense a risk to their opportunity of decision. The theory stretches out to numerous different parts of individual conduct that include motivation. This theory places that people trust they have certain freedoms and decisions and if these are threatened then contrary responses happen. At the point when practices that are seen as being free are threatened or taken away people can get to be distinctly motivated to hold and recover these freedoms. Ruth (2011).

**5.2.2 Summary of Empirical Findings**

The researcher’s findings were gotten from the hypothesis formulated and tested. It also includes a little part of her research questions which were answered by her respondents. The regression method was used to test the formulated hypothesis which involved some related question from the questionnaire administered.

1. For the first hypothesis, the relationship between the variables (compensation on employee commitment) was investigated using regression method. The result shows that there is a relationship between both variables it indicates that employees are committed to the institution through the compensation scheme.
2. The second hypothesis that examined the relationship between the variables (training and development on employee innovation) using regression method. The result show that there is a relationship between both variables.
3. Third hypothesis that has to do with the relationship between the variables (work-life balance on employee productivity) conducted using the regression method The result shows that there is a significant relationship between work-life balance on employee productivity, the null hypothesis was rejected, therefore work-life balance stimulates employee productivity.

**5.3 Conclusion**

This study concludes that there is a positive and a significant effect on motivation and employee performance. In general, compensation, training and development and work-life balance all influences the employee performance to a great extent.

Good motivating strategies generate employee performance by breaking through the competitive clutter. Motivation clearly, is an important strategy for employee performance in the institution. It is clear that motivation plays a very large role in the performance of employees, and also clear that poor motivating strategies can lead to low employee performance and lack of commitment to the institution.

From this research, it is evident that motivation cannot be isolated in practice thereby giving us the need for important strategies in order to increase employee performance. Therefore, in order to make better decisions within the institution in relation to employee performance, you need to consider and adopt motivation.

**5.4 Recommendations**

The findings of this study revealed that motivation is an important strategy for employee performance. Therefore, in the light of this, the following recommendations have been given for better effect of employee performance:

1. Institutions should adapt a non-monetary strategy for motivating employees for better performance in the work place.
2. Institutions should provide flexible working hours for their employee’s in other to enhance employee performance and productivity.
3. Since Institutions do not provide maternity and paternity leave, managers should provide their employees with both maternity and paternity leave.
4. More training and development programs should be encouraged in the institution to enhance employee innovation and creativity.

**5.4.1 Limitation of the study**

**Attitude of respondent:** unwillingness to cooperate with the study because the respondent felt they had nothing to benefit from the study whether financially or otherwise. They restricted information given due to fear of the losing their jobs.

**Coverage area:** this study was limited to only Covenant University, therefore other private universities or institutions were not adequately represented.

**Limited variables:** The study was also limited to few variables in testing the effect of motivation on employee performance.

**5.5 Suggestions for Further Research.**

There are some other areas that need to be researched on, looking at the information gathered during the research work. This study is restricted to academic staff of the institution. Therefore, in conducting further studies on this subject matter, attention should be shifted from academic staff. Other studies should focus generally on the motivating strategies in other private institution.

Furthermore, studies could focus on other motivation theories designs and could examine the design elements which enhances employee’s performance.

The method of data analysis used in this study was limited to the use of linear regression analysis. Further studies in this area can engage structural equation modelling or more detailed statistical techniques to arrive at findings that can be generalized.
